# Effect of decoration route on the nanomechanical, adhesive, and force response of nanocelluloses—An *in situ* force spectroscopy study

**DOI:** 10.1371/journal.pone.0279919

**Published:** 2023-01-03

**Authors:** Jing Li, Aji P. Mathew

**Affiliations:** Department of Materials and Environmental Chemistry, Stockholm University, Stockholm, Sweden; University of Sharjah, UNITED ARAB EMIRATES

## Abstract

Although cellulose derivatives are widely applied in high-tech materials, the relation between their force responses and their surface chemical properties in a biological environment as a function of pH is unknown. Here, interaction forces of surface modified cellulose nanocrystals (CNCs), lignin residual cellulose nanocrystals (LCNCs), and 2,2,6,6-tetramethylpiperidine-1-oxyl radical (TEMPO)-oxidized cellulose nanofibres (TCNFs) with OSO_3_^−^, COO^−^ and lignin chemical groups were measured using *in situ* peak force quantitative nanomechanical mapping and force spectroscopy in salt solution at two pH values. We found that the forces acting between the tip and CNC or LCNC are steric dominated showing long range and slow decay as a result of their low surface charge density. High Mw lignin contributed to the increased repulsion range for LCNCs compared to CNCs. The repulsion measured for TCNFs at the very short range was electrostatic force dominating showing a steep decay attributed to its high surface charge density. In the case of TCNFs, electrostatic double layer force was also evidenced by the attraction measured at secondary minima. In all the three cases the electro steric interactions are pH dependent. Dissipation maps verified that the force behavior for each material was related to structural conformation restriction of the groups at compression. The slow decayed repulsion of CNCs or LCNCs is related to a weak restriction of conformational change due to small surface groups or high molecular weight bound polymers forming flat layers, whereas the steep repulsion of TCNFs is attributed to a strong conformation restriction of carboxylic groups occurred by forming extended structure. Our results suggest that the force responses of the materials were dominated by surface charges and structural differences. TCNFs showed superior nanomechanical and repulsion properties over CNCs or LCNCs at neutral pH.

## 1. Introduction

Cellulose is a homopolymeric polysaccharide composed of β-D-glucose units linked via β-1,4 linkages. Structural advances, such as the natural hierarchical porous structure of networks, strength properties and site-specified activity, have made cellulosic materials very attractive for renewable materials with desired functions [1, 2]. Chemical oxidation of cellulose for synthesis of cellulose nanocrystals (CNCs) and cellulose nanofibers (CNFs) is an extensively used process in which the primary −OH groups at position C_6_ are oxidized to −COOH and secondary −OH groups at positions C_2_ and C_3_ to −CHO and −COOH [3–5]. The obtained materials demonstrate a chemical versatility enabled by a wide portfolio of chemical modifications on the hydroxyl groups present at their surface to render tunable surface functionality and environmental friendliness. The isolation processes for nanocelluloses mediated by sulfuric acid, phosphoric acid or organic acids, 2,2,6,6-tetramethylpiperidine-1-oxy radical (TEMPO)-mediated oxidation etc. introduces anionic surface functionalities as -OSO_3_^−^, -PO_4_^3-^ or COO^−^ groups on nanocellulose surface. These anionic surface groups can interact with positively charges entities like metal ions, dyes etc. as adsorbents [6]. In the field of water treatment, we have recently used *in situ* peak force quantitative nanomechanical mapping (PFQNM) combining with nanocellulose-functionalized colloidal probes to reveal mechanisms of the enhanced adsorption properties of functionalized nanocelluloses, TEMPO-oxidized CNFs (TCNFs), OSO_3_-CNCs and residual lignin-grafted CNCs (LCNCs) in the presence of water pollutants in aqueous condition [7–11]. Charge specific attraction or repulsion is considered as the main mechanism of pollutant removal using the nanocellulose based adsorbents. Our results demonstrated that high surface charge and reduced surface roughness rendered the better functions, compared to non-modified surface of nanocelluloses [12]. Zwitterionic polymer-grafted CNCs provided a pH-specific surface charge for the adsorption of ions [13].

Although it is clear that decreasing the charge density of the surface will result in a reduction of the adsorbed amount of e.g. heavy metal ions, it is still unclear on their force responses of the aforementioned nanocelluloses at nano scale in a biological environment as a function of pH. The salt concentration of the solution in which the adsorption takes place is an important factor that controls the swollen cellulose structure, the assembly kinetics, and assembling particles as strength additives [14]. The association between the charged chemical groups on nanocelluloses and charged compounds like salt ions in electrolytes at interfaces is very complicated, because we have to consider interaction forces between the interface and the ions, as well as between the interface and the charged CNFs or CNCs [15].

In this respect, a current challenge is therefore to understand how the surface chemical properties and structures of the functional groups (-OSO_3_^−^, -PO_4_^3-^ or COO^−^) influence force response of nanocellulose surfaces and especially in use condition(liquid environment and varying pH condition). This will be essential for understanding how to best tailor the materials from the nanoscale components using modified production processes [16–18]. Till now, no systematic study has elaborated the points.

Among a few promising *in situ* methods for tackling the challenge, it is of advantage that atomic force microscope (AFM) based measurements can be conducted in aqueous conditions, which provide *in situ* information of properties of the materials during real time interaction between two surfaces [19]. PFQNM is an excellent AFM imaging mode to extract localized mechanical properties by monitoring force-distance curves at each image point, and to acquire images of elasticity, adhesion and other mechanical properties while producing high resolution morphology maps at the same time. Comparing conventional tapping mode, the advantage of peak force tapping mode is that tip-sample interaction is extracted one point on the sample within an acquisition time of typically a second, using a triggering sinusoidal excitation that is much lower than resonance frequency of a cantilever [20, 21].

Force spectroscopy using an AFM has been widely used to quantify interaction forces of cellulosic colloidal systems in liquid conditions. The defined natures of force have provided many insights on the functioning mechanism of the system during interfacial reactions [22–28]. The primary information obtained from force measurements is the force acting between two macroscopic surfaces as a function of surface separation [29]. The interplay between attractive van der Waals forces and repulsive double layer forces forms the basis of the classical Derjaguin–Landau–Verwey–Overbeek (DLVO) theory to explain the smooth interface such as particle-solution with a well-defined boundary. Both of these forces have been directly measured between various surfaces [30, 31]. The double layer repulsion, unlike the van der Waals attraction, is much more sensitive to the type and concentration of electrolyte present, the pH, and the surface charge density or potential. Deviations from classical DLVO theory exist and the force may be attractive before it becomes repulsive; for e.g. in polymer systems, complex molecular rearrangements and other interactions can lead to quite complex resultant forces [32, 33].

Here, using PFQNM and force spectroscopy, we aimed to study the force response of the OSO_3_^−^-modified CNCs, -COO^−^-modified TEMPO oxidized TCNFs, and residual lignin CNC (LCNCs) in a biologically relevant environment in a salt electrolyte at two pH values, and to clarify the relations between the force response and the surface properties of three types materials. We hypothesized that a better understanding on how surface properties of the materials, including crystallinity, surface charge density, modifying group coverage have affected on their localized nanomechanical properties, force behaviors as a function of pH is required for optimization of the performance of the nanocelluloses such as adsorption and antifouling.

## 2. Materials and methods

### 2.1. Materials

The characterized materials were acquired using the methods in our previous studies [11, 13]. In short, OSO_3_^−^ modified CNC (CNCs) suspensions (5 wt. % in water, crystallinity degree 76.3 ± 5.6%) were purchased from CelluForce, Canada. The CNCs were isolated using sulfuric acid hydrolysis and carried anionic charge on the surface. Lignin residues grafted CNC (LCNCs) were synthesized in our lab (1.4 wt%, crystallinity degree 64.8 ± 2.0%) [34]. LCNCs were prepared using partial bleaching for the residual production of bioethanol from wood containing 50% (w/w) cellulose, 42% (w/w) lignin, and 8% (w/w) extractives using high-pressure homogenization. TEMPO oxidized COO^−^ modified CNFs (TCNFs) (1.1 mmol/g carboxyl groups, 2.0 wt.%, crystallinity degree 60%) were provided by Swiss Federal Laboratories for Materials Science and Technology, Switzerland [35]. TCNFs were produced by surface carboxylation of CNFs using TEMPO as the catalyst and subsequent mechanical disintegration. TCNF surfaces were partially carboxylated and hence negatively charged. All three nanocellulose materials possess a high surface-to-volume ratio and high surface charge density with negative charges. Their surfaces are intrinsically hydrophilic owing to the presence of hydroxyl and carboxyl groups. Crystallinity of the samples was determined by X-ray diffractometer (XRD). The surface charges were determined by Zeta potential measurements. Experiment procedures in detail, contact angle and Zeta potential data for the three materials can be found in SI 1.4 in S1 File.

### 2.2. Cantilever calibration and PFQNM measurements

PFQNM experiments were conducted using a Bruker Dimension Fast-scan instrument. Prior to each measurement in liquid, a ScanAsyst-fluid AFM probe (tip radius Nom: 20 nm; Spring constant, Nom: 0.7 N/m; Tip material: silicon nitride) was treated with a UV ozone cleaner (ProCleaner Plus) for ca. 20 min prior to use. The deflection sensitivity of the AFM detector was obtained against a hard surface (Sapphire calibration kit) and then the cantilever spring constant (*k*) was calibrated using the thermal tuning method, as developed by Hutter and Bechhoefer [36], using the built-in option in software Nano scope version 9.1 [20]. The end tip radii of the probes we used were determined to be in the range 10~14nm. This was determined in two ways, by utilizing a polycrystalline titanium roughness sample or measuring the elastic modulus on a reference sample (a blend of polystyrene and polyolefin elastomer) with known modulus. See S2 Table.

Prior to PFQNM measurements, diluted nanocellulose suspensions were immobilized on freshly cleaved mica with pre-deposited (3-aminopropyl)triethoxysilane(APTES) monolayers [7]. All *in situ* PFQNM experiments were carried out in phosphate-buffered saline (PBS) buffer salt electrolytes with a constant salt concentration (150 mM) at two pH values, neutral 7.2 and acidic 3.5. PBS buffer was chosen with the purpose of simulating a relevant biological environment. PBS pH 7.2 (150 mM) was purchased from Sigma. PBS pH 3.5 solutions were freshly prepared with deionized water (Milli-Q System). The pH was set to pH 5 using acetate and to pH 7 by phosphate and to a constant ionic strength of 150 mM at pH 7.2. All chemicals used were of analytical grade. Each nanocellulose sample was measured at two different pHs 3.5 and 7.2 respectively. Please note that the samples were not dried between the two pHs. The data presented here is on never dried sample. For each material, one sample was measured at pH 3.5, and another sample was measured at pH 7.2. Measurements were conducted in triplicate using calibrated ScanAsyst-fluid probe. A detailed scheme for AFM sample preparation is found in S1-S1.5 in S1 File.

To ensure that the AFM probe was pressed sufficiently hard onto the surfaces to obtain reliable force curves in air and in aqueous solutions, the deformation depth was carefully controlled during each PFQNM measurement and was found to be in the range of 5.0~17.0 nm. The speed of the probe approaching the surfaces was set to 0.1 Hz with 150 nm peak force amplitude and 2 kHZ peak force frequency in all cases of the measurements. The approach speed (nm\s) varies in a sinusoidal fashion according to the principle of peak force controlled tapping mode, but can be calculated from the amplitude and frequency. A maximum of 512 × 512 pixel force was recorded for each mapping, and more than 2 × 10^5^ FD curves were acquired over a full round of force mapping. Every interaction between the tip and sample is analysed, and from this, the mapping of the modulus, adhesion force, dissipation, indentation, and topography of the surface is simultaneously obtained. Individual force curves at each pixel were extracted. Of note, the force curves obtained at a certain experimental condition were highly reproducible over many consecutive compression cycles and that all force curves obtained using the same condition showed the same trend. The shape of the interaction curves was unaffected even after leaving the samples up to 8 hours immersed in the salt solutions at either one of the two pH conditions tested. See S3 Fig. The FD curves displayed in result section are only representative force curves showing characteristic force behaviors of each of the material. The Zeta potential data in S1 Table has the relation with the measured force according to linear Poisson-Boltzmann equation [37].

### 2.3. PFQNM analysis

The tip surface force during PFQNM measurement is described in Eq 1. *F*−*F_adh_* is the force on the cantilever relative to the adhesion force, *R* is the tip end radius, and *d*−*d*_0_ is the deformation of the sample. The result of the fitting is the reduced modulus E*; if the Poisson ratio is known, Young´s modulus (E_s_) of the sample will be calculated by the software.


$$F-F_{adh}=\frac{4}{3}E^{*}\sqrt{{R(d-d_{0})}^{3}}$$ (1)

The Derjaguin-Muller-Toporov (DMT) model implemented in the Nanosope Analysis (V2.0) software was used to obtain the modulus and adhesion force mapping during the PFQNM measurements, where the localized Young´s module (denoted as modulus or DMT modulus in the following texts) and adhesive force at any interesting location of a PFQNM image were calculated. The DMT model includes adhesion outside the contact area and is suitable for high elastic moduli, low adhesion and a small radius of indentation and was proven to be suitable for nanocellulose studies [19, 38]. In our case, it is a safe approximation because indentations are small, adhesion is weak in aqueous environments, and there is an acceptable difference of ca. one order of magnitude in the curvature of cellulose surface compared with the AFM tip.

The statistics of the distribution of the modulus and mapped adhesive force were fitted using height and slope distribution function and Gaussian fitting implemented in Gwyddion program, which is a powerful AFM data analysis platform [39–41]. Normalization of the densities *ρ* (*p*) (where *p* is the corresponding quantity) is described by the equation *f*_*(x)*_ *= y*_*0*_ *+ a exp[−(x − x*_*0*_*)*^*2*^*/b*^*2*^*]* implemented in the program [42]. These functions are computed as normalized histograms of the height, force or modulus (obtained as derivatives in the selected direction –horizontal or vertical) values. Hence, the scale of the values is independent of the number of measured data points and the number of histogram buckets. The cumulative distributions are integrals of the densities, and they have a value of 0 or 1. The fitted values of height, modulus, adhesion force and dissipation of the PFQNM mappings obtained at their peak density of the Gaussian fitting curves and fitting procedure are shown in the S2 File, S3 Table, S4 Fig, respectively.

## 3. Results and discussions

**Fig 1:**
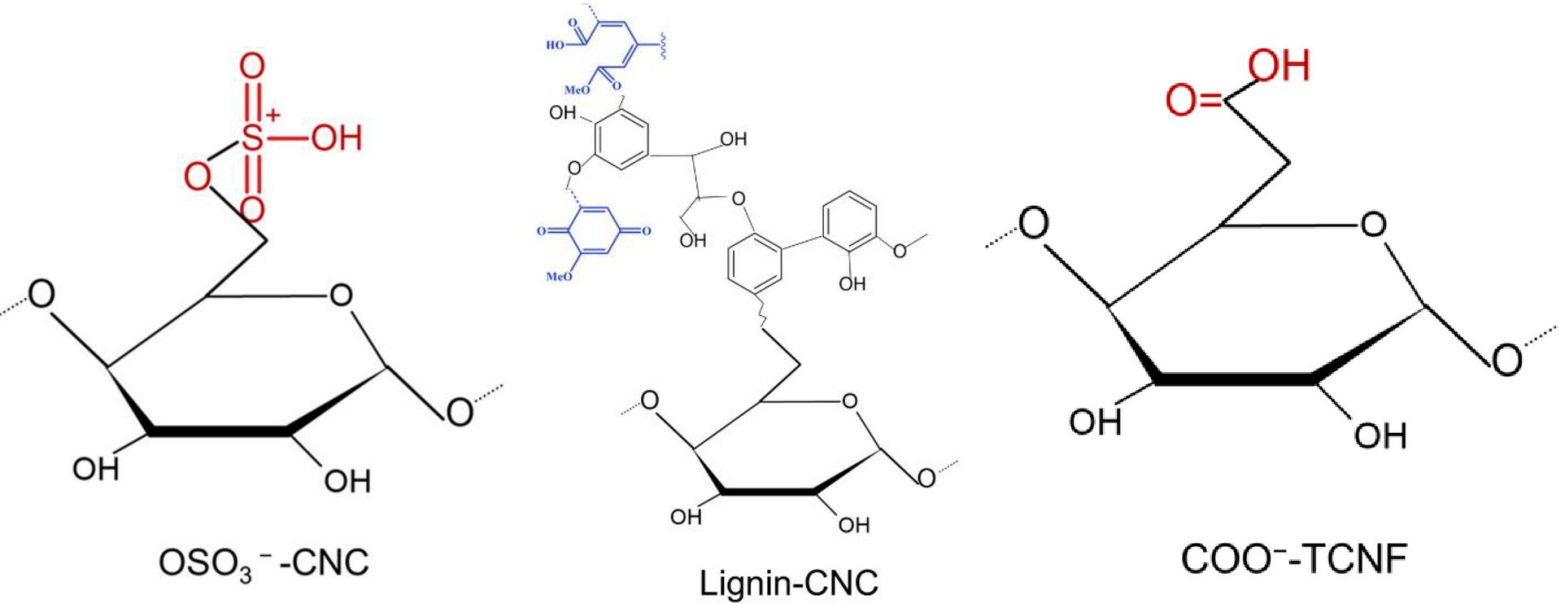
Schematic illustration of the structures of the decorating OSO_3_^−^ (H^+^)groups on CNCs, lignin groups on CNCs, and COO^−^(H^+^) groups on TCNFs. Exact protonated or deprotonated form of the group will depend on the pH condition.

First of all, the AFM probe used in this study is made by silicon nitride. The tip charges at pH 3.5 and pH 7.2 are negative, and it is expected that the charge remains roughly constant during the measurements [43]. As illustrated in Fig 1, the structures of the modified nanocelluloses are composed of bounding chemical groups of COO^−^, OSO_3_^−^ or lignin residues owning groups of COO, CHO^−^ and phenolic C_10_H_12_O_3_ on the nanocellulose backbones. In addition, XPS and NMR data obtained by us demonstrated successful grafting of chemical groups on the three materials: O-SO_3_ groups on CNCs, the COO^−^(H^+^) groups on TCNFs, and the residual lignin groups on LCNCs [11, 12]. The data supports the schematic diagram of the structures in Fig 1.

### 3.1. Nanomechanical properties of the nanocelluloses

**Fig 2:**
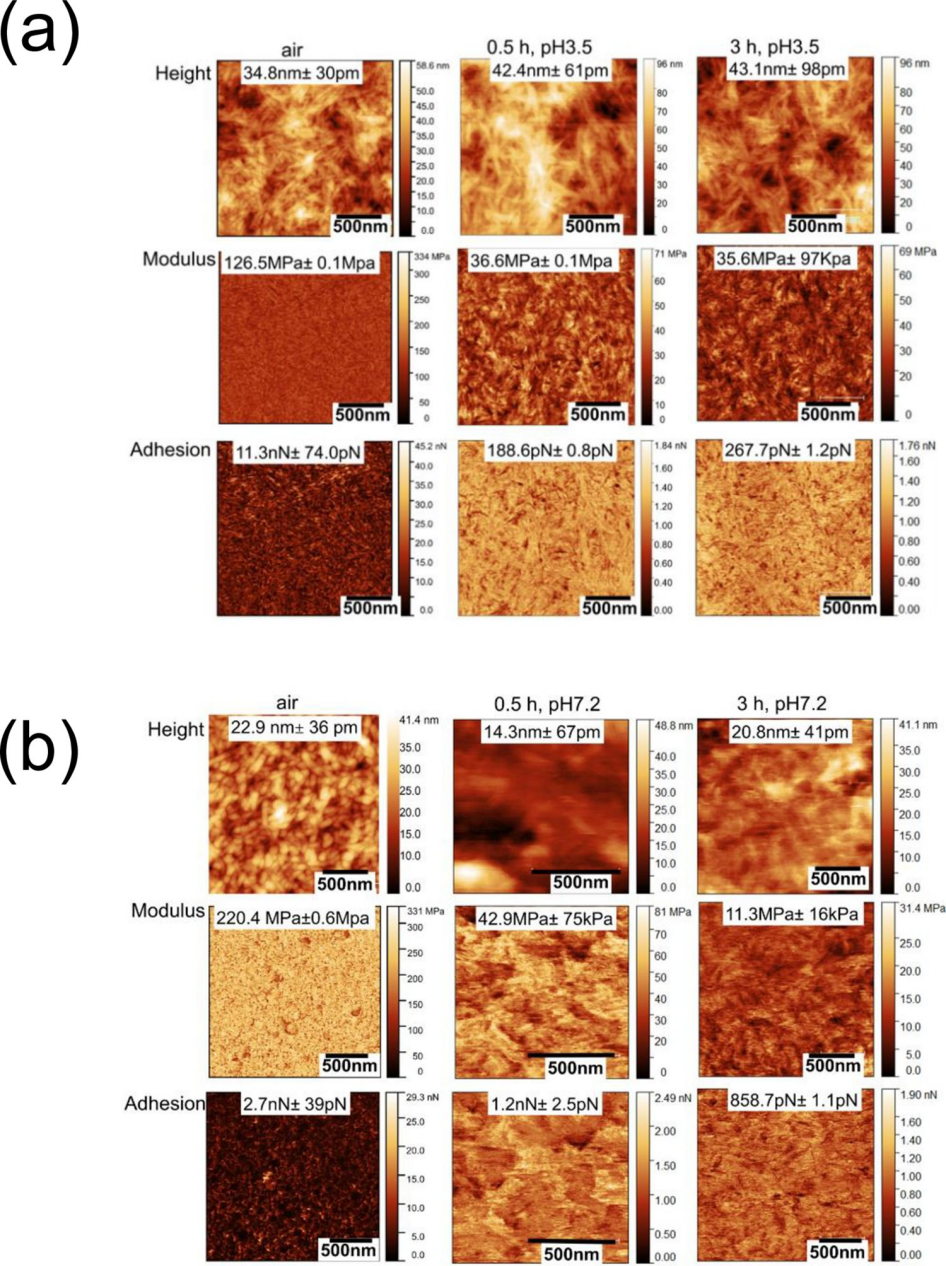
Sequential PFQNM images of CNCs obtained in air and then in PBS buffer after 30 minutes after 3 hours of immersion at pH 3.5 (panel (a)) and pH 7.2 (panel (b)). (scale bar: 500 nm).

**Fig 3:**
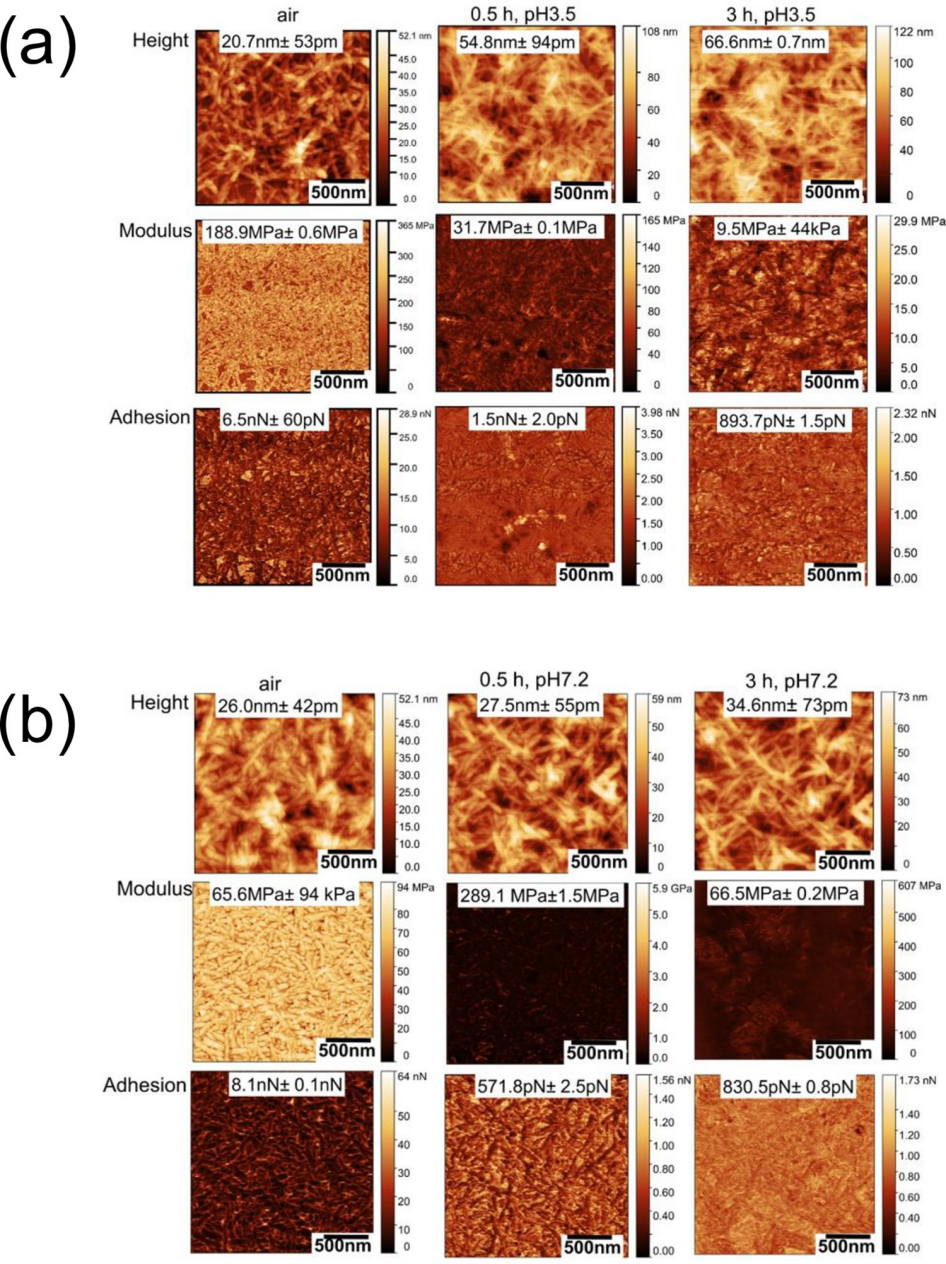
Sequential PFQNM images of LCNC obtained in air and then in PBS buffer after 30 minutes after 3 hours of immersion at pH 3.5 (panel (a)) and pH 7.2 (panel (b)). (scale bar: 500 nm).

**Fig 4:**
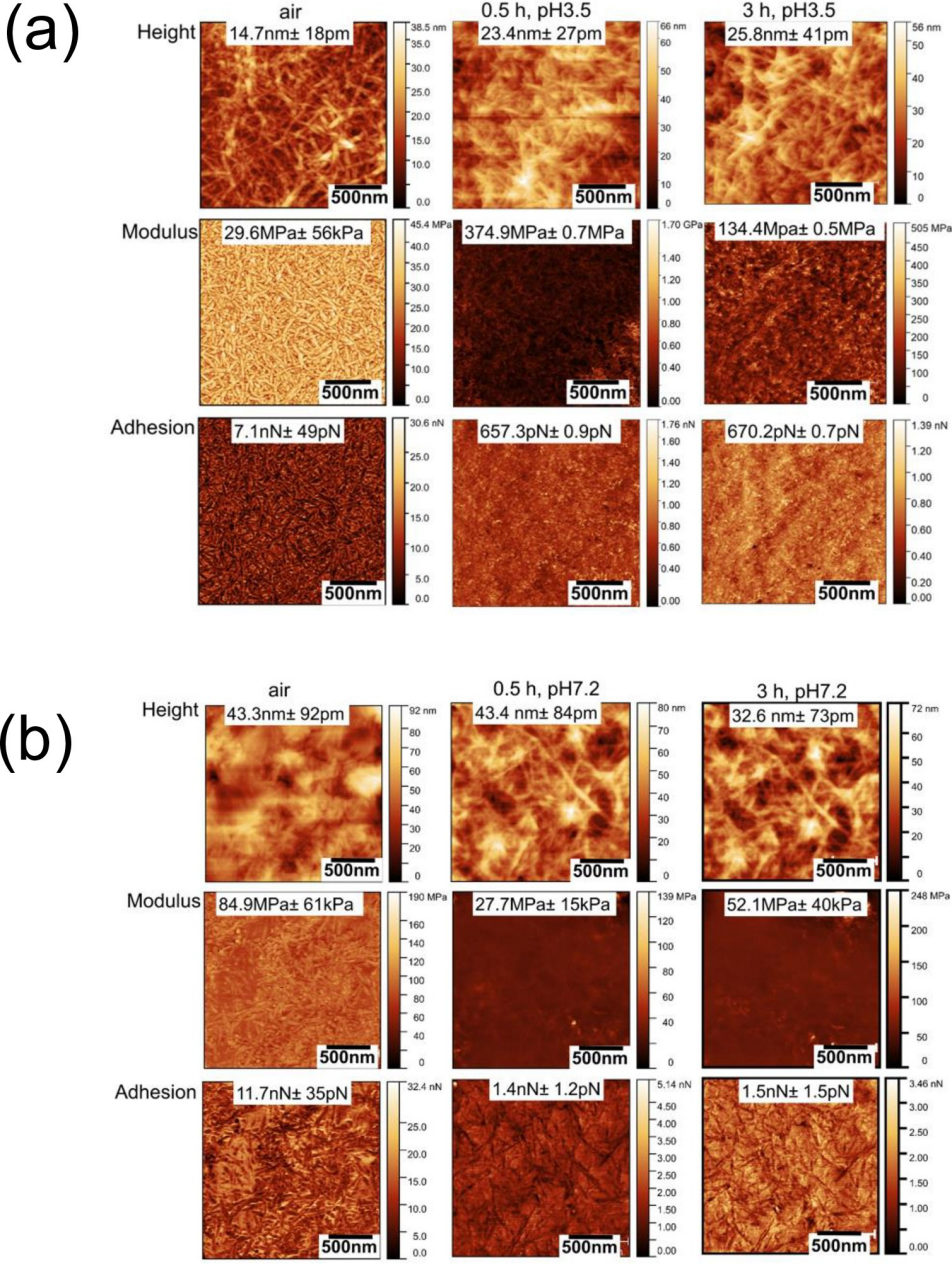
Sequential PFQNM images of TCNFs obtained in air and then in PBS buffer after 30 minutes after 3 hours of immersion at pH 3.5 (panel (a)) and pH 7.2 (panel (b)). (scale bar: 500 nm).

Figs 2–4 show sequential PFQNM images of morphology (height), modulus, and adhesion force mapping of the CNC, TCNF and LCNC samples, respectively, in air and in PBS electrolytes after 30 minutes and 180 minutes of immersion at either of the two pH values (pH 3.5 and pH7.2).

In all cases the network structure of nanocellulose was clearly visible in air and in liquid medium. The modulus and adhesion force were higher in air than that in electrolytes for all the studied materials. For all the three cases, statistic data of the modulus or adhesion force showed time and pH dependence. The modulus of CNCs decreased with time where as in the case of TCNFs the modulus at both pHs. The modulus of LCNCs increased with time at pH 7.2, while it decreased at pH 3.5. The TCNFs possess the highest surface charge density at pH 3.5 followed by the CNCs and LCNFs. The surfaces charges directly impacts the interaction forces and modulus [44]. The decreased modulus of CNCs and LCNCs at pH 3.5 maybe was caused by water adsorption due to the hydrophilic nature of these materials. In contrast, the sustained modulus of TCNFs is related to its high surface charges adsorbing the salt ions competing over the water penetration [8]. The difference in the charge density is negligible between the three materials, at pH 7.2. All three materials are negatively charged as proved by the Zeta potential data in S2 Fig.

As shown in Figs 2 and 3, TCNFs and LCNCs showed better stability (stable modulus values) in salt solution at both pH values and superior properties in terms of nanomechanical strength than CNCs. The higher elastic modulus for TCNFs and LCNCs was attributed to ion adsorption by the negative charges of COO^−^ and lignin residues, leading to an increased “conjunction” at fibre contacts of the nanocellulose networks [45]. We observed a decrease in the modulus for CNCs at both pH values, which was attributed to the ion-induced water uptake. It was reported that the structural differences between the fibrillar networks however impact their degree of swelling. The cellulose networks can plastically deform upon swelling driven by ion-induced osmotic pressure [44]. The sustained modulus of TCNFs was also attributable to crosslinking of the TCNF networks in presence of salt ions overtaking the decrease in modulus due to water adsorption as reported by us [46].

The strength of the adhesion force is in the order of TCNFs > LCNCs > CNCs, at both pH. The adhesion forces measured for the TCNFs at neutral pH is two fold higher (at nN) than that measured at the acidic condition (pN level), showing a significant pH dependence. The highest adhesion forces measured for TCNFs is related to the high surface charge and high availability of binding sites at individual TCNF. This can also be attributed to its architectural advantage − high aspect ratio, uniform widths and nanodispersed architecture, thanks to the TEMPO oxidization [5]. The adhesion force of LCNCs increased from 571 pN to 830 pN after three hours of immersion at pH 7.2. The results clearly indicate that the surface charges causes strong adsorption of salt ions such as K^+^ and Na^+^. Our result agreed with our previous *in situ* PFQNM results that an increase in stiffness for TCNFs occurred under neutral condition due to due to bounding of metal ions Cu(II) to the carboxylate groups [47, 48]. Another observation is that despite that the low surface charges of LCNCs in acidic pH condition, LCNCs still gave rise to a higher adhesion force than that measured for CNCs at pH 3.5. The result indicated that high contact areas of lignin residues at the LCNCs provided active sites for adsorbing salt ions. This is evidenced by the fact that the average size of a LCNC single fibre was approximately 50 nm larger than a CNC as shown in line profiles for randomly selected single fibres (see S6 Fig). A large area of LCNC surfaces was also reported in our previous study [11].

The elastic modulus and adhesion force results demonstrated that the surface charges and structural advantages of the TCNFs have played significant roles in improving the nanomechanical/ion adsorption properties. The low surface charged and less organized structure of the CNCs or LCNCs are not in favor of maintaining their modulus in salt solutions. The pH dependence of the averaged adhesion forces further confirmed the importance of the surface charges affecting on nanomechanical properties of the three materials, as agreed with our previous results obtained by other characterisation methods. Because the differences in hydrophilic properties of their surfaces are negligible, the modulus and adhesion properties are related the charges and structures of the nanocellulose network rather than hydrophobicity [49]. In addition, one should be cautious when comparing modulus results between one study and another. As that the variation of the modulus values can be large and perhaps not fully comparable due to the differences in the geometries of AFM tips employed, indentation depth of AFM tip against the surface and the modulus modelling [50]. Therefore, our results are meaningful for a comparative study [51]. The elastic modulus results also agreed with previously reported PFQNM values [48, 52].

**Fig 5:**
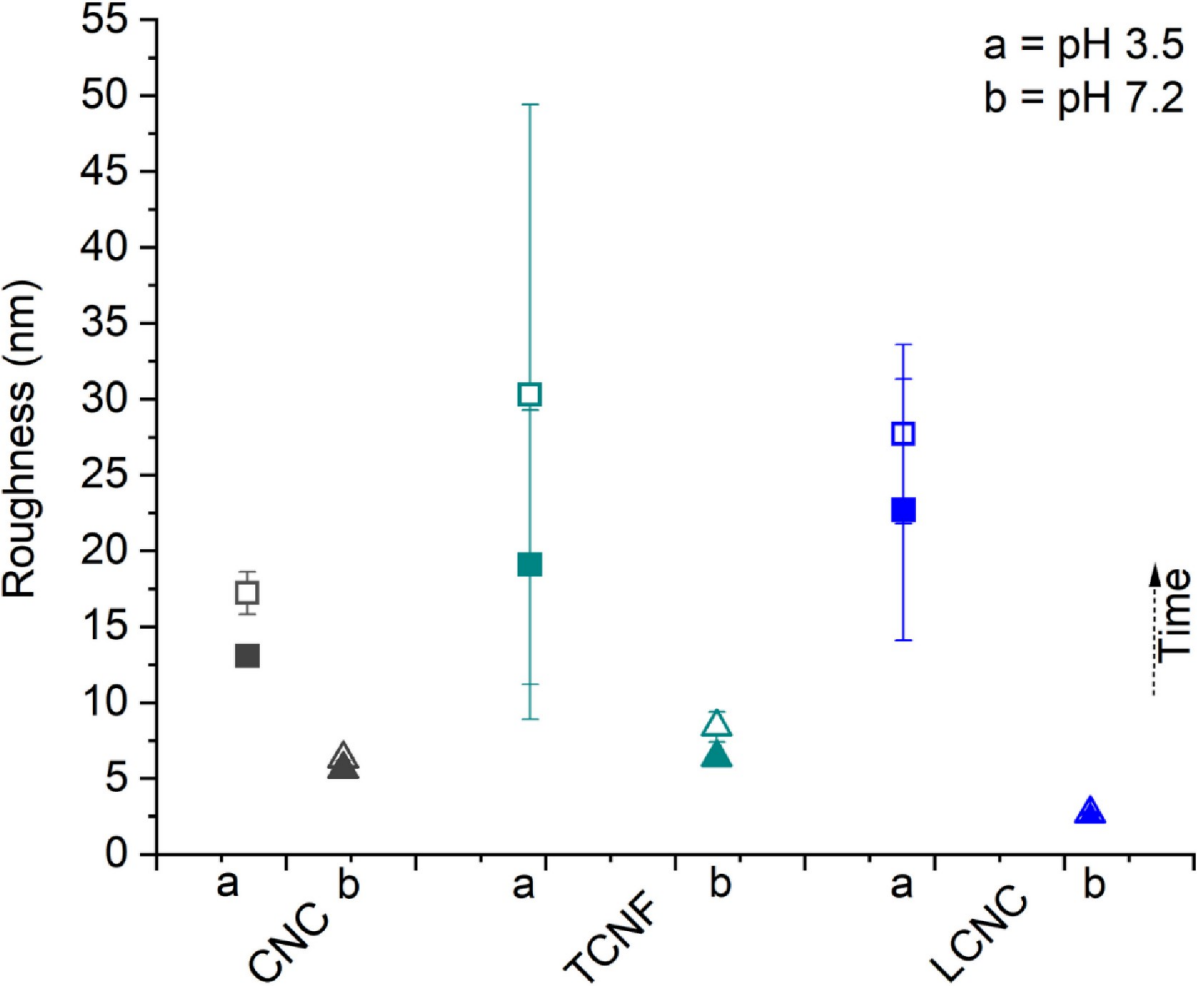
Comparison of the roughness values of the nanocellulose surfaces obtained at pH 3.5 and 7.2 as a function of immersion time.

Fig 5 and S4 Table show statistical values of the roughness (500 nm × 500 nm) of the nanocelluloses. The values were calculated using the same way of analysis as that conducted for the PFQNM results shown in Figs 1–3. The roughness of the film samples is higher at pH 3.5 than that at pH 7.2 for each case. LCNCs is the roughest followed by TCNFs and CNCs at pH 3.5; the roughness measured at pH 7.2 is in the order of TCNF > CNC > LCNC. The changes in roughness with time of immersion are more significant at pH 3.5 than that observed at neutral pH 7.2. The result may indicate that the surfaces of nanocelluloses tended to readily crosslink in the presence of monovalent (Na^+^, K^+^) ions at neutral pH, leading to a smoother surface, as has been previously reported by us [53].

### 3.2. Repulsion and attraction forces at tip compression − force origin and its relation with surface chemical properties of the nanocelluloses

**Fig 6:**
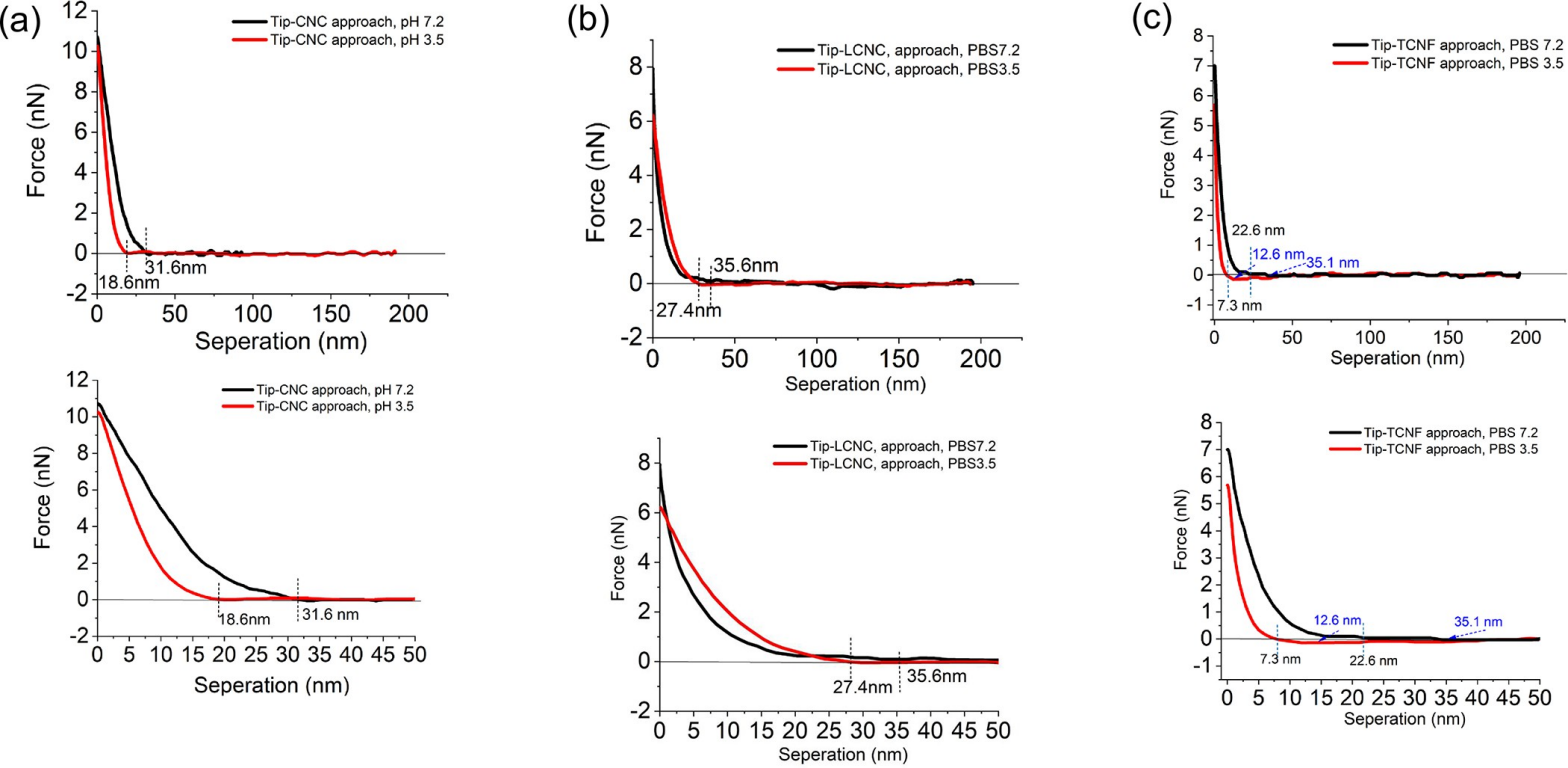
Representative force-distance approach curves recorded at a single (a) CNC, (b) LCNC, and (c) TCNF fibre measured at pH 3.5 and 7.2.

The force distance (FD) curves in Fig 6 distinguished the differences in terms of the separation range, steepness and magnitude of the force interactions measured for deprotonated (pH 7.2) and protonated (pH 3.5) chemical groups for each type of the nanocelluloses. Of note, instead of seeking a suitable model to fit the force at specific separation distances in detail, we only have focused on comparative discussions on the force profiles and their differences measured between the three types nanocelluloses.

As shown in Fig 6(A)–6(C), the repulsion measured for CNCs and LCNCs at tip approach are evidenced at relatively long ranges of separation- i.e., 18.6 nm for CNCs, and 27.4 nm for LCNCs at pH 3.5. Under neutral condition at pH 7.2, the repulsions were measured at separation of 31.6 nm for CNCs and 35.6 nm for LCNCs. The repulsion range is longer at neutral condition for each case showing a clear pH dependence. The repulsion range measured for CNCs is shorter than that for LCNCs at either pH. The repulsion measured for the TCNFs was at range of 7.3 nm at pH 3.5 and at 22.6 nm at pH 7.2, respectively. The repulsion range is thus the longest for LCNCs followed by CNCs, and the shortest for TCNFs.

The FD curves measured on approach were plotted on a logarithmic scale force vs. separation distance for direct analysis (see S5 Fig). The logged force curves showed significant differences in steepness at the separation distance where the repulsion force was raised for each case. In principle, at higher ionic strength in monovalent electrolytes, the double layer repulsion usually remains strong enough to keep the surfaces apart; this is because the van der Waals attraction is weak. A typical electrostatic double layer repulsion in a monovalent electrolyte solution shows rough exponential decay at the separation below the Debye length. In our case, the salt concentration used for the force spectroscopy measurements is 150 mM\L, Debye length for 1:1 electrolyte at 298 K (25°C) is only 0.8 nm. See S3 File [30]. This is also in agreement with reported Debye length of TCNF elements, which is in the range of 0.68 ~0.96 nm in NaCl solution at concentrations of 100–200 mM [54]. As we can see, the repulsion measured for CNCs and LCNCs at approach decayed much slowly as separation decreased. The repulsion decay for TCNFs increased more steeply than that for CNCs and LCNCs. All three repulsion is not an exponential decay, and is lying out of the Debye length.

Therefore, our FD data shows deviations from the DLVO theory. The repulsion are not attributable to pure electrostatic double layer forces. Though, most experimental deviations in the forces from those expected from the DLVO theory are not due to any breakdown in the DLVO theory, but rather to the existence of a stern-layer or to the presence of other forces such a ion-correlation, solvation, hydrophobic, or steric forces [30]. Moreover, as shown in S3 Fig, the curves (deflection error vs. Z distance (nm)) measured at approach are always more repulsive than those determined on separation. This hysteresis is a strong evidence that the forces measured are dominated of steric origin [55]. We concluded that the repulsion measured for all three cases consists of steric force and electrostatic force components [26, 30, 56–60].

As shown in Fig 6(C), noticeably, secondary minima attractions were measured for TCNFs at a longer range separation at either of the pH value. The attractions are evidenced at the separation of 12.6 nm at pH 3.5, and of 35.1 nm at pH 7.2, before the appearance of the repulsion. Interestingly, the electrostatic attraction is more distinguishable as measured at pH 3.5 than that at pH 7.2. The attraction appeared at the longer range at pH 7.2 than that at pH 3.5, showing a clear pH dependence. In principle, the secondary minima is a characteristic salt/pH dependent minima, which is attributed the reduction of surface charge density. To conclude, the repulsive deviation from DLVO theory observed in all three cases, suggesting a steric origin of repulsion, adding the fact that this component of the force curve is sensitive to the surface charges of the nanocellulsoes given the significant pH effect we observed. We termed the repulsions measured for all three materials as an “electro-steric” component. Mechanisms of the origin of force for each case are discussed further down. In addition, as shown in S5 Fig, the force measured for the TCNFs at the long range (> 50 nm) separation displayed recognizable attraction at tip retraction at either pH. The occurrence of attractions for the CNCs and LCNCs over the course of tip retraction is not distinguishable, and the measured forces are mostly repulsive; Therefore, the retraction force profiles for the two CNCs are not discussed herein.

The force behaviors studied using force spectroscopy for biopolymer colloidal systems largely depend on the structure of the polymers and experimental conditions [30, 61]. The force profiles observed in all three cases as shown in Fig 6(A)–6(C) suggested steric force and electrostatic force origins. We consider this to be electro-steric repulsion, which is well agreed with previous report [62]. P. Claesson et al., reviewed that charged polyelectrolytes adsorb to similarly charged surfaces only if the non-electrostatic affinity is sufficient to overcome the electrostatic repulsion and the entropic penalty of adsorption. Thus, polyelectrolyte adsorption occurs easily at high salt concentrations such like in our case the salt concentration of 150 mM is thought to be high, and in presence of low charge density polyelectrolytes, generating long-range repulsive forces of electro-steric origin [15].

The decaying steepness (i.e. slope of the approach curve above force zero) and ranges of the forces measured for the cellulose nanomaterials at approach showed highly pH- and charge-dependence, which is related to surface charge density and molecular weight (Mw) of the groups (COO^−^, OSO_3_^−^ or lignin residues), structure layers, and crystallinity [30, 56].

In the cases of CNCs and LCNCs, the very slow decay of the repulsion at tip approach demonstrated that steric force are the dominating component in the total measured force. It was reported that slowly decaying repulsion usually occur during a steric repulsion in aqueous solution when a polymer possesses low surface charges, high chain density, and high crystallinity. The forces are usually created by a moderate/small restriction of the conformation of the polyelectrolyte surfaces [61, 63, 64].

Another observation is that, at either pH condition, the range of the repulsion force measured for LCNCs was created over a larger distance than that for CNCs, suggesting that there likely was a high degree of dissociation of the lignin, and that the lignin residues were stretched away from the nanocrystal backbone. In fact, the distance of separation values marked in Fig 6(B) indicated that the lignin groups have extended approximately 13.7 nm at pH 3.5 and 17.8 nm at pH 7.2 into solution at approach, since the repulsion force occurs in the inner regime (separation distance < 50 nm) at the separation range twice the values at either the pH condition [62]. The repulsion range is shorter at neutral pH than acidic pH for either of the case, showing a clear pH dependence. Therefore, the measured repulsions must contain electrostatic double layer forces [62].

Interestingly, moreover, when we compared the separation ranges of LCNCs and CNCs obtained at the two pH conditions, as shown in Fig 6, we found that there is no significant change in the repulsion ranges measured for LCNCs, whereas there is an obvious change in repulsion range measured for CNCs. This suggests that the electrostatic repulsion between lignins and the tip surface are capable of competing with the Van der Waals attraction force regardless its surface charge density. Whereas the range of repulsion measured on CNC depends on pH, which perhaps is due to its slightly high surface charge density.

By considering the Mw of the groups on the surfaces CNC or LCNC materials as that the difference in surface charge of the two materials is small (see Zeta potential data), the negligible change in the separation ranges observed for the LCNCs evidently suggested that the surface of lignins are in an expanded state, and lack of conformation restriction due to the high Mw of lignin. Whereas, the small Mw OSO_3_^−^ groups bounded on the surface of the CNCs perhaps encountered moderate to large conformational restriction giving rise to the shorter ranged repulsion. Therefore, we concluded that the slow decay and steric dominated repulsion measured for the two cellulose nanocrystals are attributed to the fine architecture of the crystalline CNC and LCNC and the high density of the chemical groups at the nanocrystal backbones, as well as the high Mw lignin and low Mw OSO_3_. The high Mw lignin residues must have formed a thick flat layer at the tip compression giving rise to the force measured at a longer range of separation [15, 59, 62, 65].

As for the force behaviors of TCNFs, it was reported that uniformly charged polysaccharide such as TEMPO oxidized TCNFs repelled from the other negatively charged surface, due to the steric and double layer repulsions between the two similarly charged surfaces. They can also be attracted to each other depending on the relative strengths of the van der Waals and electrostatic forces [62, 63, 66]. Therefore, we assumed that the TCNFs have a sufficient amount of side groups with the charges (COO^−^) to counteract attraction, and at the same time enough electrostatic attachment points at its half-left sites of the fibres to ensure a strong attraction to another surface [59, 67, 68].

Such type of force behaviors are clearly observed in the force profile measured for the TCNFs. It is well known that only half of the accessible hydroxymethyl groups of TCNFs are available to oxidation into COO^−^ [69]. As shown in Fig 6(C), a steeply increasing repulsion force was measured for TCNFs at the shortest range of separation, and the range of repulsion was significantly longer in pH 7.2 than that measured in pH 3.5. The pH dependence of the repulsion ranges is a clear evidence showing that electrostatic force plays dominating roles in the measured total force during the charging (de-protonation) process of the COO^−^ group at the high pH. The relatively longer ranged attraction force in Fig 6(C) has been assigned to the characteristic salt/pH dependent secondary minima due to charge effects. We clearly observed that the repulsion component of the total measured force was suppressed because the reduced amount of charges of TCNFs during the protonation process at acidic condition of pH 3.5, making the double layer attraction force more significantly observable than that measured at pH 7.2. Interestingly, the strong effect of the charges of the TCNFs on the electrostatic double layer attraction was further evidenced by the pH dependence of the attraction ranges at the minima region, where the separation range of the attraction at pH 3.5 is shorter due to the largely reduced charges.

It was reported that, in highly concentrated electrolyte solutions, a significant secondary minimum usually appears beyond 3 nm, before the energy barrier close in. The minima should only appear when the surface charge density has been sufficiently reduced by e.g. protonation bring about by decreasing the pH, according to the DLVO theory [30]. The secondary force minimum comes from a disturbance on the balance of forces, where the van der Waals electrostatic force contributes the attractive component. The minimum appears when the repulsive force is sufficiently suppressed as displayed in the force behaviors for the case of TCNFs, it is this reduction caused the attractive van der Waals electrostatic double layer force being easily observable at the secondary minima region. Taken together, we concluded that the force measured for TCNFs at approach is electrostatic force dominated, compared to CNCs or LCNCs. In contrast, no attraction at the secondary minima was measured for either CNCs or LCNCs at approach, which is probably due to their low charge density in comparing to TCNFs.

Given the structure advantage of the TCNFs, the possible causes for generating the short ranged steep repulsion and minima attractions can a proper ratio between the highly charged COO^−^ groups and CNF segments on TCNFs created by the highly discriminative TEMPO oxidation method. On the one hand, the steeply raised repulsion came from that the sufficiently charged COO^−^ groups tended to form a well-extended structural conformation and to be firmly balanced at the tip surface, leading to rough exponential repulsive energy per unit area and thus the steep decay [15]. This has happened and it is due to that each of the sided isolated COO^−^ interacts independently of the other groups, because there is no overlap of neighboring groups as a result of its optimal surface coverage of TCNFs. On the other hand, the attraction can be due to that some unoccupied surface sites at the other half of the inaccessible hydroxymethyl groups were left to facilitate the overlapping of the groups, thus generating van der Waals attractive force at a certain distance of separation [30, 66, 70–72].

In addition, as shown in the FD curves in S5 Fig, the appearance of attraction force measured for TCNFs at tip retraction is attributed to strong electrostatic screening on the charges of the groups causing ionic adsorption [45, 71, 73]. The negatively charged surface of TCNFs is cross-linked by monovalent counter salt ions (K^+^, Na^+^) at the patches on the cellulose fibres, leading to attractive forces [61]. The attraction has driven the ion adsorption, leading to the increased elastic modulus of the TCNFs. This is correlated to the results in section 2.1. The differences in force behaviors measured for the threes materials are essentially related to conformational changes of the structure layers of the chemical groups caused by energy dissipation at compression. The underpinning mechanisms are further discussed in the section 2.3 below.

### 3.3. Dissipation and conformational restrictions of the groups on nanocelluloses affect on the interaction forces

**Fig 7:**
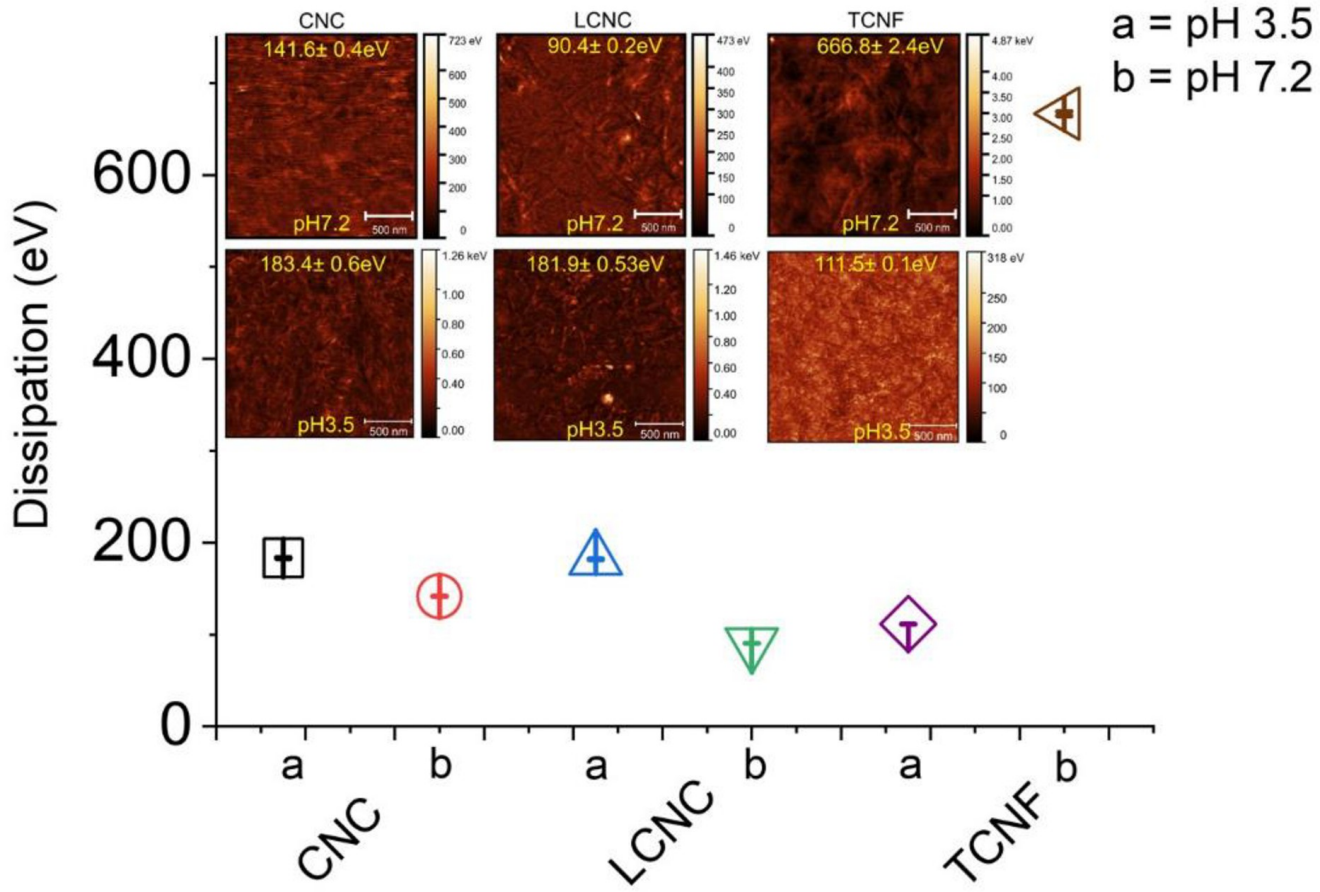
Fitted dissipation values and dissipation maps for CNCs, LCNCs and TCNFs obtained at pH 3.5 and pH 7.2 respectively. (scale bar: 500 nm).

Fig 7 shows the fitted dissipation values obtained at peak position measured from the Gaussian fitting for each material, and the inserted images are the dissipation maps for each material obtained at the either of the two pH values. At pH 3.5, the energy loss that occurred at CNCs is largest 183.35 ± 0.62 eV, followed by 181.86 ± 0.53 eV for LCNCs, and 111.46 eV ± 0.13 eV for TCNFs. At pH 7.2, the dissipation measured for TCNFs is the highest 666.8 ± 2.40 eV. In contrast, small energy loss was measured for the nanocrystals, which is 141.62 ± 0.44 eV for CNCs, follows by 90.436 ± 0.16 eV for LCNCs. The dissipation measured for all three cases at pH 7.2 differed significantly whereas it was roughly constant at pH 3.5, presumably as the materials possess higher charges groups at pH 7.2.

The changes of structural conformation of the three materials are quantitatively verified by the dissipation results of PFQNM, as plotted in Fig 7. In principle, the origin of the repulsive force between two similarly charged surfaces in a solvent containing counterions and electrolyte ions is entropic (osmotic), not electrostatic. On bringing two such surfaces together, one is therefore forcing the counterions back onto the surfaces against their preferred equilibrium state −that is, against their osmotic repulsion but favored by the electrostatic interaction [30]. In our case, the system is rather complex where both counterions and electrolyte ions exist. We assumed that the chemical groups are protruding far out into the electrolyte solution, their protrusions become increasingly confined into a smaller region of space under a compression, and, the surface groups can dissociate the counterions (H^+^, Na^+^, K^+^) at the compression. When the two surfaces get close, the chemical groups rapidly experienced an enforced restriction of conformation, causing the disturbance of equilibrium state of the surfaces due to the electrostatic force. It is this disturbance that causes a significant loss of entropy (i.e. the measured dissipation), the restriction of the conformation will give rise to repulsive forces [30, 45, 56, 57, 61, 62, 71, 74–78]. How much the chemical groups that are bond to the surfaces extend out from these creates smaller or bigger steric restriction when the two surfaces approach one and another. This determines the magnitude and steepness of the repulsion force generated [15, 65].

The small dissipation measured for CNCs and LCNCs indicated the formation of flat structure of the groups preventing group interdigitating, due to their high crystallinity and low surface charge density [15, 26, 59]. As a result, the repulsion force showed the slow decay. The dissipation measured for TCNFs is much larger than the ones for CNCs and LCNCs at pH 7.2, suggesting that a more strong restriction of conformation occurs for TCNFs. The deprotonated carboxyl groups experience strong restriction associating with an extended conformation during interdigitating at the tip compression. Moreover, the measured dissipation for TCNFs at pH 7.2 is much stronger than that found at pH 3.5. These variations was attributed to the ionization (charging) of the COO^−^ groups on the surface of TCNFs at the higher pH. This forces the charged groups to adopt a more extended conformation. Because of this, the measured repulsion range at neutral pH is significantly longer (22.6nm) than that (7.3nm) obtained at acidic condition when the groups are protonated [15]. The secondary minima attraction is also related to conformational changes of the chemical groups attached on the surface of TCNF material [30]. It might be due to that the highly charged COO^−^ or COOH groups at either pH stand more upright or extend out, and can collapse more in the opposite case, bringing the underlying surfaces closer to the surface of AFM tip and therefore making the van der Waals attractive force easier observable.

Taken as a whole, the above results demonstrated that the repulsion and attraction behaviors for all three cases is of steric and electrostatic force origins at approaching and retraction. Clear differences in the force response due to different layer structures on a molecular level were observed between highly charged, low Mw TCNFs, lowly charged low Mw CNCs and lowly charged high Mw LCNCs.

Steric repulsion dominated forces were measured for the lower charged CNCs or LCNCs at tip approach. At either pH, the high Mw LCNCs formed a thick layer giving rise to steric force at longer range than that for CNCs, whereas low Mw CNCs gave rise to shorter ranged steric repulsion attributing to slightly higher charges of CNCs. In either case, longer range repulsion was measured in pH 7.2 than that in pH 3.5 showing a pH dependence, suggesting that electrostatic force plays roles in the total measured repulsion forces but not acting as a dominating component. In contrary, the steep slope of the repulsion measured for TCNFs indicated that the measured repulsion is of an electrostatic force dominating origin.

The appearance of the secondary minima attraction measured for TCNFs and its pH dependence at approach demonstrated that the electrostatic double layer force plays main role for the total measured force attributing to its high surface charge density. The dominating effect of the surface charge density of TCNFs on the force response was further verified by the very long ranged attraction measured at tip retraction as a result of electrostatic screening on the highly charged carboxyl groups. The measured repulsion forces are termed as an electro-steric repulsion for all cases.

The force behaviors of the three materials are related to their structural differences, which is verified by energy dissipation data. The strong dissipation measured for TCNFs suggested that strong conformation restriction at COO^−^ groups occurred. And probably it was due to that an extended layer of the carboxylic groups were formed at tip compression as a result of the high charge density and TCNFs´ structural advantage where the ratio between the charged COO^−^ groups and active interacting sites of the TCNF segments is optimized. The weak dissipation measured for CNCs or LCNCs suggested that somehow flat layers were formed during interactions between the OSO_3_ or lignin groups bound on the nanocrystals and similarly charged tip surfaces. The conformation changes are associated with the low surface charges, high crystallinity of the two types of cellulose nanocrystals, and high Mw lignin.

We have been cautious with regards to making exact definitions of the forces at long-range separation (> 50 nm) due to the use of the sharp AFM tip. Given that DLVO or non-DLVO models only work well for interaction radii much larger than the separation distance, this may not be true in our case. It is therefore reasonable that we do not define the forces exactly. For all three materials, though we observed that there is a balance between attractive and repulsive forces in most cases at long-range separations. We concluded that the very long-range interaction forces indicate some degree of steric interaction at tip compression. This means that the fibre could have been winded up (reversible) in a secondary minimum and flocculated (reversible aggregation) [79].

## 4. Conclusions

In this work, the localized nanomechanical properties, adhesive force, and interaction force origins of surface modified nanocellulose with OSO_3_^−^, COO^−^, lignin residual groups were directly measured using PFQNM and force spectroscopy with a sharp AFM tip in a salt electrolyte at two pHs.

TCNFs displayed better nanomechanical properties than that CNCs and LCNCs as demonstrated by the sustained and increased modulus during immersion at either pH condition. The force profiles quantitatively measured the forces behaviors between the negatively charged nanocellulose surfaces and the similarly charged surface of the AFM tip, revealing the relations between the force origins and the surface chemical properties of the materials. We found that the measured force are of electro- steric origin in all three cases. The repulsion force measured for CNCs and LCNCs at approach are of a dominating steric origin attributing to their low surface charge density and high crystallinity. High Mw lignin contributed to the longer range of separation than that for CNCs. The repulsion measured for TCNFs is dominated by electrostatic force because of its high surface charges. The longer ranged attraction force measured for TCNFs at secondary minima and its pH dependence further verified that electrostatic force dominated the total measured force attributable to its high surface charge density.

The differences in force behaviors are related to conformational restriction on the chemical groups during compression as revealed by the dissipation maps. We found that the strong conformation restriction of carboxylic groups on TCNFs occurred causing the steeply raised repulsion, which is attributed to optimized side chain and charge density ratio of TCNFs. Whereas the weak conformational restrictions occurred on the groups of the cellulose nanocrystals leading to slow decayed repulsion, attributable to their low surface charges and less optimized structure layers.

It is concluded that the high surface charge density and optimized ratio of charged groups and fibre segments of the TCNFs made by TEMPO oxidization method has largely contributed to its better performances in terms of nanomechanical strength and interaction force performance for adhesion of salt ions and perhaps repulsion against biomolecule adsorption in a biological relevant environment in comparing with the performances of the cellulose nanocrystals. Overall, our results clarified the relationship between the elastic modulus, interaction force, nanostructures and surface chemical properties of the three materials as a function of pH, suggesting that desired functionalities of surface modified nanocelluloses require a careful consideration of chemical treatment route using a bottom-up approach. In addition, the methodology can be applied to interactions between nanocellulose and more complex biological objects such as proteins or even bacteria under relevant experimental conditions. Our findings may be relevant in the field of functionalized cellulose nanomaterials for a range of applications.

## Supporting information

S1 File Experimental details for material synthesis, protocol of the experimental setups and measurements. (DOCX)

S2 File A short description of the height and angle distribution function in Gwyddion program. (DOCX)

S3 File A brief description of the calculation of Debye length (*λ*_D_). (DOCX)

S1 Fig Representative photographs of the water drop captured during a contact angle measurement for each material. (DOCX)

S2 Fig Zeta potential results of the nanocellulose sample suspensions obtained in PBS buffer solutions at pH 3.5 and 7.2, respectively. (DOCX)

S3 Fig Images and representative deflection error (nm) vs- Z distance (nm) curves measured on two single fibers of the same CNC sample. (DOCX)

S4 Fig Representative results of a Gaussian fitting for the (a) height, (b) DMT modulus, (c) adhesion force, and (d) dissipation mapping of PFQNM images of the nanocelluloses obtained in PBS solution.
(DOCX)

S5 Fig The logged FD curves of (a) CNC, (b) LCNC and (c) TCNF as displayed in the main text of Fig 6. The retraction force curves of the (d) CNCs, (e) LCNCs and (f) TCNFs plotted together with the approach curves are presented in Fig 6. Deflection error vs. Z distance (nm) curves measured on each of the marked single fibre of the three materials at pH 7.2 or 3.5.
(DOCX)

S6 Fig Line profile of a randomly chosen area of each of the DMT modulus mapping (drawing a horizontal and a vertical crossing line over an image) and adhesion force distribution (drawing a line over a single fibre) of the PFQNM images of (a, a´) CNCs, (b, b´) TCNCs and (c, c´) LCNCs obtained in air and in PBS buffers. Representative line profiles of height and width of a single fibre of CNC, TCNF, LCNC obtained in ambient (air) condition, by randomly chosen five single fibres on the same sample.
(DOCX)

S1 Table Information on the surface chemical properties of the nanocelluloses in PBS buffer solutions. (DOCX)

S2 Table Representative values of the calibrated spring constant of ScanAsyst-Fluid cantilevers prior to each PFQNM measurement in PBS buffer. (DOCX)

S3 Table Fitted values of distribution of the height, DMT modulus, and adhesion force mappings displayed in Figs 2–4 in the main text. (DOCX)

S4 Table Root mean square roughness (*Rq*) values of the height mappings (500 nm × 500 nm) of PFQNM results as a function of immersion time. (DOCX)

S1 Graphic abstract (DOCX)
