# Supplementary material for: Effect of decoration route on the nanomechanical, adhesive, and force response of nanocelluloses—An in situ force spectroscopy study
Source: PLoS One. 2023 Jan 3;18(1):e0279919. doi: 10.1371/journal.pone.0279919 (PMC9810197; doi:10.1371/journal.pone.0279919)
Supplement: S1 File — (DOCX) [file pone.0279919.s001.docx]

**Supplementary information (SI)**

**S1 Information: experimental details for material synthesis, protocol of the experimental setups and measurements;**

SI 1: experimental details for material synthesis, protocol of the experimental setups and measurements

**SI 1.1.** Preparation of sulfate modified CNCs

The OSO_3_^−^ modified CNCs in the laboratory was achieved via sulfuric acid hydrolysis. Dry sheets of sulfite softwood cellulose, kindly provided by Aditya Birla Domsjö, were used as starting material. 100 g of dry cellulose sheets were dispersed in 1 L of a 64% sulfuric acid solution by strong mechanical stirring at 45°C. After 45 minutes, 10 L of deionised water were added in order to quench the reaction and the dispersion was allowed to sit for approximately three hours.

**SI 1.2.** Preparation of TCNFs by TEMPO-mediated oxidation

TCNFs were prepared following a similar protocol to the one established by Saito and Isogai. [1] The never-dried pulp was preliminarily washed using hydrochloric acid (pH = 2) in order to remove impurities from the pulping process. 40 g of never-dried pulp was oxidised with a ratio of 10 mmol of sodium hypochlorite (Merck) per gram of pulp over 200 minutes at pH = 10, in the presence of TEMPO (0.64 g, Sigma-Aldrich) and sodium bromide (4 g, NaBr, Sigma- Aldrich). The oxidized pulp was then washed multiple times with deionized (DI) water until the conductivity of the filtrate fell below 5 μS.cm^-1^.The TEMPO-mediated oxidation was followed by a mechanical grinding treatment using a supermass colloider (Model MKZA10-15 J, Masuko Sangyo Co., Ltd.). The oxidised pulp, diluted to a concentration of 0.5 wt. %, was fed four to five times through the grinder’s non-porous silicon carbide grinding stones (Disk model MKE), which were rotating at a speed of 25 Hz and separated by a gap clearance of 50 μm, resulting in a homogeneous TCNF gel-like dispersion.

**SI 1.3.** Preparation of LCNCs

### LCNCs were made by bleaching and subjected to mechanical treatment through a high-pressure homogenizer. The residue from the production of bioethanol was subjected bleaching processes to obtain a partly bleached material.  For the partially bleached material, 30 g of pulverized extractive free residue was added with 10 g of NaClO_2_ and 4 mL of CH_3_COOH in 2 L of H_2_O. The reaction mixture was stirred for 1 h at 70 °C, and then equal amounts of NaClO_2_ and CH_3_COOH were added. This is considered as one bleaching cycle, and it was repeated two more times (in total, 30 g of NaClO_2_ and 12 mL of CH_3_COOH were added during 3 h). After the last bleaching cycle, the material was washed with H_2_O via centrifugation..[2] Nanocrystals were prepared from the the partially bleached materials (also termed lingo residued nanocrystals (LCNCs),) by subjecting each of them to mechanical treatment through a high-pressure homogenizer. Prior to homogenization, solutions of ∼1% (w/w) were prepared and sonicated with a titanium rod sonifier (QSonica sonicators, Q500) at 24 kHz for 10 min to avoid the presence of large aggregates. Dispersions were then passed 10 times through an APV 2000 high-pressure homogenizer at a pressure of 600–800 bar. After homogenization, the obtained nanocrystals were dialyzed in Milli-Q water by using a 14000 Da molecular weight cutoff dialysis membrane (Sigma-Aldrich) for one day.

**SI 1.4.** Zeta potential measurements

The surface charges were determined by Zeta potential measurements. The experimental details are, in short, a Zetasizer Nano ZS (Malvern Panalytical Ldt.) was used to assess the Zeta potential of the nanocellulose dispersions. Three 5ml samples of the suspensions in PBS salt solutions and in equal to the nanocellulose concentrations as the same as that for making AFM samples on mica were prepared. When used, a part of the samples was poured into a DTS0012 cuvette, and a universal ‘dip’ cell kit (ZEN1002) was then carefully placed into it. Each sample was measured five times in total. Furthermore, each measurement consisted of 10 repeated cycles. The calculated standard deviation of every five measurements was adopted as the error of each sample.

### SI 1.5. The scheme below shows the procedure for preparation of the samples on mica, and the experimental setups for PFQNM measurements in liquid. There was a fluid meniscus (ca. 200 uL electrolyte drop) formed prior to a PFQNM measurement, and it remained between the probe holder and the mica during the entire period of time of testing hours.

###
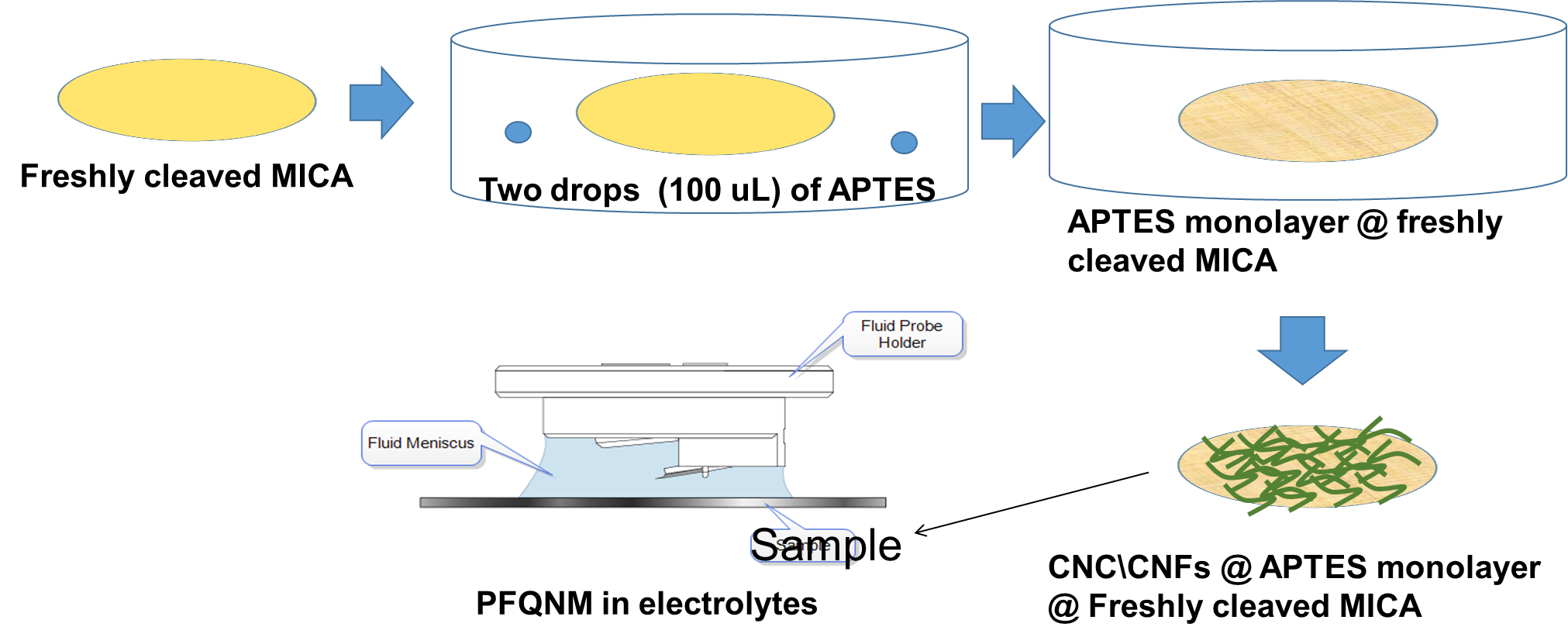


**SI 1.6**. Below shows two typical photographs of the samples taken during a PFQNM experiment after withdrawing the scanner of the Fast Scan Head. As can be seen, the electrolyte drops remained on top of the mica. The results evidently showed that the samples were immersed in electrolytes during the entire period of time of AFM measurements. The PFQNM modulus, force curve data were thus acquired in electrolyte solution throughout the measurements for each material at each of the pH condition.

**
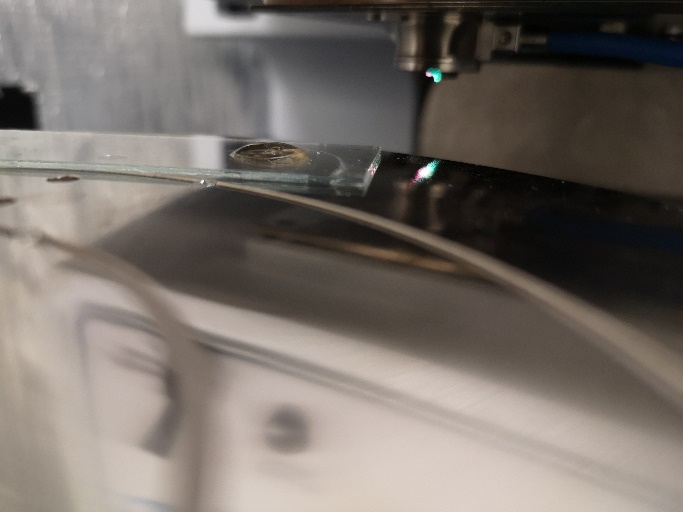

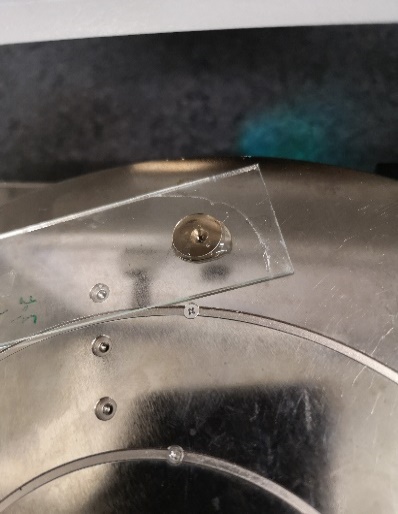
**

Electrolyte drop

Moreover, we want to point out that the samples were not dried between the two pH, pH 3.5 and pH7.2. For PFQNM measurements, each nanocellulose samples was measured at the two different pHs. The data presented here is on never dried sample. For each material, one sample was measured at pH 3.5, and another sample was measured at pH 7.2.

The representative AFM images below were acquired in air for the CNCs, TCNFs and LCNCs immobilized on mica after they were heavily rinsed by DI water after several hours of PFQNM measurements in pH7.2 or 3.5 salt solutions. As we can see, for all three cases, the nanocellulose are observable after up to 10 hours of immersion in the electrolyte at either ph7.2 or 3.5.


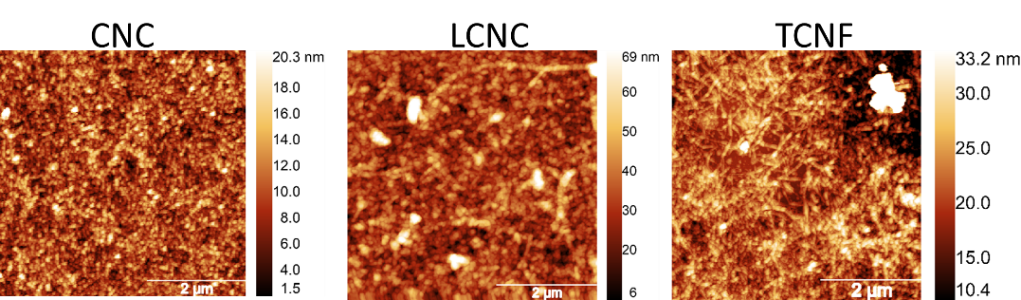


Figure. Representative AFM height morphology of (left) CNCs, (middle) LCNCs, and (right) TCNFs obtained in air after conducting PFQNM measurements in electrolyte pH 7.2.

For each of the material, nanocellulose are not only clearly observable but also well remained at the mica surface. We can conclude that, firstly, the images evidently approved that the nanocelluloses were still remained very well at the end of each of our PFQNM experiments for all three types of materials. This indicates that the sample preparation procedure is reliable. Second, we are assured that the APTES monolayers were completely covered by the nanocellulose fibres. As a result, the high coverage of the nanocelluloses can certainly rule out any uncertain effects from the under-layer of APTES on the nanomechanical and force results measured for the nanocellulose surfaces per se.

**References**

1. Saito T, Isogai A. TEMPO-Mediated Oxidation of Native Cellulose. The Effect of Oxidation Conditions on Chemical and Crystal Structures of the Water-Insoluble Fractions. Biomacromolecules. 2004;5: 1983–1989. doi:10.1021/bm0497769

2. Georgouvelas D, Jalvo B, Valencia L, Papawassiliou W, Pell AJ, Edlund U, et al. Residual Lignin and Zwitterionic Polymer Grafts on Cellulose Nanocrystals for Antifouling and Antibacterial Applications. ACS Appl Polym Mater. 2020;2: 3060–3071. doi:10.1021/acsapm.0c00212
