# Supplementary material for: Effect of decoration route on the nanomechanical, adhesive, and force response of nanocelluloses—An in situ force spectroscopy study
Source: PLoS One. 2023 Jan 3;18(1):e0279919. doi: 10.1371/journal.pone.0279919 (PMC9810197; doi:10.1371/journal.pone.0279919)
Supplement: S2 File — (DOCX) [file pone.0279919.s002.docx]

**Supplementary information (SI)**

**S7 Information: A short description of the height and angle distribution function in Gwyddion program;**

Height and angle distribution function in Gwyddion, functions are computed as normalized histograms of the height or slope (obtained as derivatives in the selected direction – horizontal or vertical) values. Using equation

f(x) = y_0_ + a exp[−(x − x_0_)^2^/b^2^]

The normalization of the densities *ρ*(*p*) (where *p* is the corresponding quantity, height or slope) is such that

$$\int_{-\infty}^{\infty} \rho\left( p \right)dp=1$$

Evidently, the scale of the values is then independent on the number of data points and the number of histogram buckets. The cumulative distributions are integrals of the densities and they have values from interval [0, 1].
