# Supplementary material for: Effect of decoration route on the nanomechanical, adhesive, and force response of nanocelluloses—An in situ force spectroscopy study
Source: PLoS One. 2023 Jan 3;18(1):e0279919. doi: 10.1371/journal.pone.0279919 (PMC9810197; doi:10.1371/journal.pone.0279919)
Supplement: S3 File — (DOCX) [file pone.0279919.s003.docx]

**Supplementary information (SI)**

**S12 Information: A brief description of the calculation of Debye length (*λ*_D_).**

At large distances, this electrostatic double-layer force decays roughly exponentially. The decay length is the Debye length.[1] For a monovalent salt it is

$$\lambda D=\sqrt{\frac{\varepsilon\varepsilon_{0}k_{B}T}{2Cⅇ^{2}}}$$

The Debye length *λ*_D_ is determined by the salt concentration; *C* is the salt concentration in mol/L. For water at 25 °C, the equation can be reduced to λ_(D)_ =3.04/c Å. If ions of higher valency *Z_i_* are also present, 2*c* has to be replaced by ∑ciZi2. Here, *c_i_* is the bulk concentration of the ion species. The sum runs over all ions present. *k_B_* is Boltzmann constant (1.381 × 10^−23^ J/K).

**Reference**

1. Israelachvili JN. Intermolecular and Surface Forces. Elsevier; 2011. doi:10.1016/C2009-0-21560-1
