# Supplementary material for: Effect of decoration route on the nanomechanical, adhesive, and force response of nanocelluloses—An in situ force spectroscopy study
Source: PLoS One. 2023 Jan 3;18(1):e0279919. doi: 10.1371/journal.pone.0279919 (PMC9810197; doi:10.1371/journal.pone.0279919)
Supplement: S1 Fig — (DOCX) [file pone.0279919.s004.docx]

**Supplementary information (SI)**

**S2 Fig. Representative photographs of the water drop captured during a contact angle measurement for each material;**


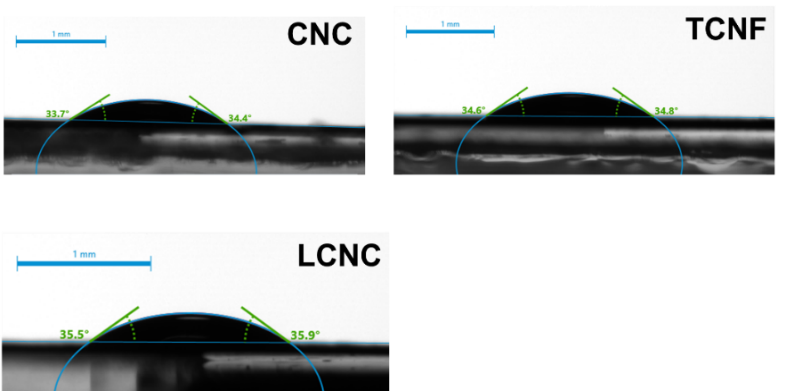


**Fig S2**
