# Supplementary material for: Effect of decoration route on the nanomechanical, adhesive, and force response of nanocelluloses—An in situ force spectroscopy study
Source: PLoS One. 2023 Jan 3;18(1):e0279919. doi: 10.1371/journal.pone.0279919 (PMC9810197; doi:10.1371/journal.pone.0279919)
Supplement: S2 Fig — (DOCX) [file pone.0279919.s005.docx]

**Supplementary information (SI)**

**S3 Fig. Zeta potential results of the nanocellulose sample suspensions obtained in PBS buffer solutions at pH 3.5 and 7.2, respectively;**

**Fig S3**

As can be seen in Fig S3, the surfaces of TCNF are the most negatively charged. The surfaces of LCNF are the slightest negatively charged. By changing the pH of solution from pH 7.2 to 3.5, zeta potential of LCNCs decreased more strongly than that of CNCs and TCNFs.
