# Supplementary material for: Effect of decoration route on the nanomechanical, adhesive, and force response of nanocelluloses—An in situ force spectroscopy study
Source: PLoS One. 2023 Jan 3;18(1):e0279919. doi: 10.1371/journal.pone.0279919 (PMC9810197; doi:10.1371/journal.pone.0279919)
Supplement: S3 Fig — (DOCX) [file pone.0279919.s006.docx]

**Supplementary information (SI)**

**S6 Fig. Images and representative deflection error (nm) vs- Z distance (nm) curves measured on two single fibers of the same CNC sample.**


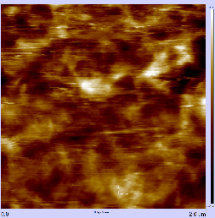

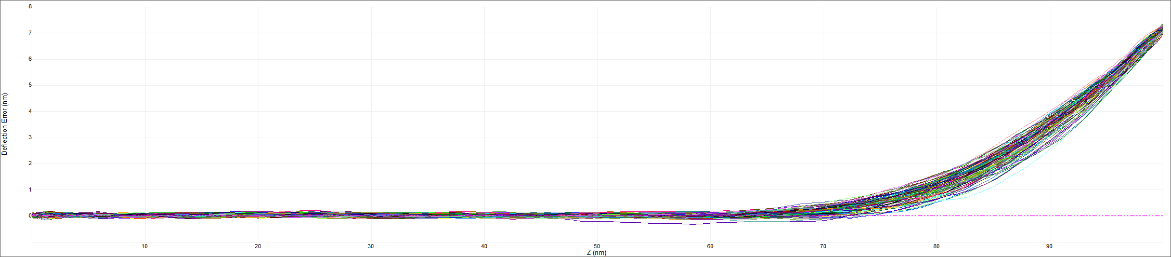


CNC pH7.2


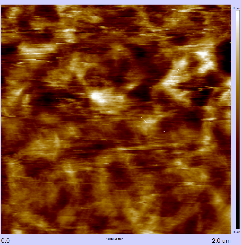


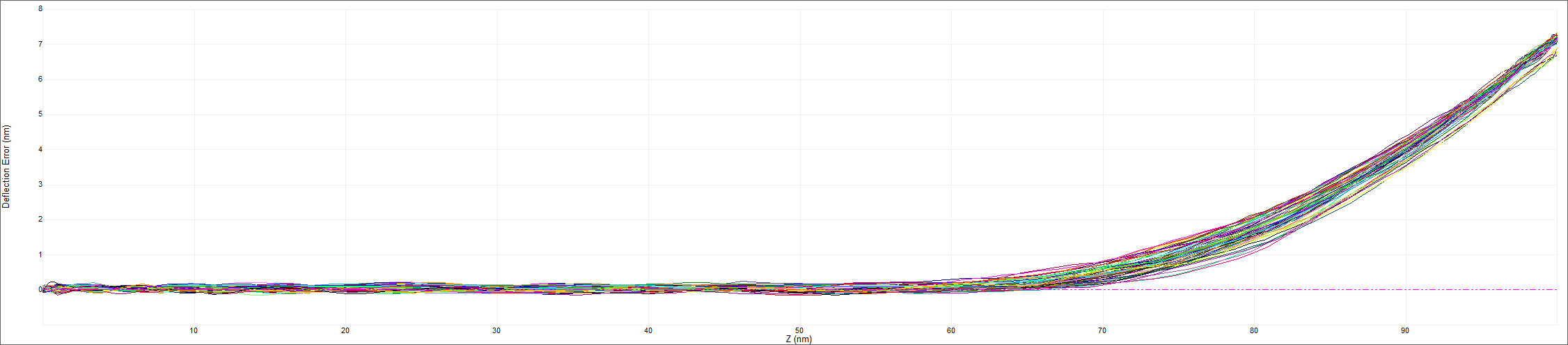


CNC pH7.2

**Fig S6**

Representative deflection error (nm) vs. Z distance (nm) curves measured on a single fiber of CNCs are shown in Fig S6. The images show an examples that the measurements of many more Deflection error (nm) vs. Z distance (nm) curves were conducted for two single fibres of the CNC nanocelluloses. The measurement was done by simply drawing a line crossing on top of the single fibre (as marked by the blue arrow). Noticeably, the force curves obtained at a certain area of a single fibre were highly reproducible, and that all force curves obtained using the same pH condition for each type of the samples showed the same trend. The shape of the interaction curves was unaffected even after leaving the samples immersed in the salt solutions up to 10 hours at either the two pH conditions tested. Therefore, we think that the statistical data reported for the modulus, adhesion force and dissipation, and the analysis for the highly repeatable force interaction data should give high statistical accuracy. The method used for statistical analysis in this work is considered to be reliable and statistically significant.
