# Supplementary material for: Effect of decoration route on the nanomechanical, adhesive, and force response of nanocelluloses—An in situ force spectroscopy study
Source: PLoS One. 2023 Jan 3;18(1):e0279919. doi: 10.1371/journal.pone.0279919 (PMC9810197; doi:10.1371/journal.pone.0279919)
Supplement: S4 Fig — Representative results of a Gaussian fitting for the (a) height, (b) DMT modulus, (c) adhesion force, and (d) dissipation mapping of PFQNM images of the nanocelluloses obtained in PBS solution. (DOCX) [file pone.0279919.s007.docx]

**Supplementary information (SI)**

**S9 Fig. Representative results of a Gaussian fitting for the (a) height, (b) DMT modulus, (c) adhesion force, and (d) dissipation mapping of PFQNM images of the nanocelluloses obtained in PBS solution;**


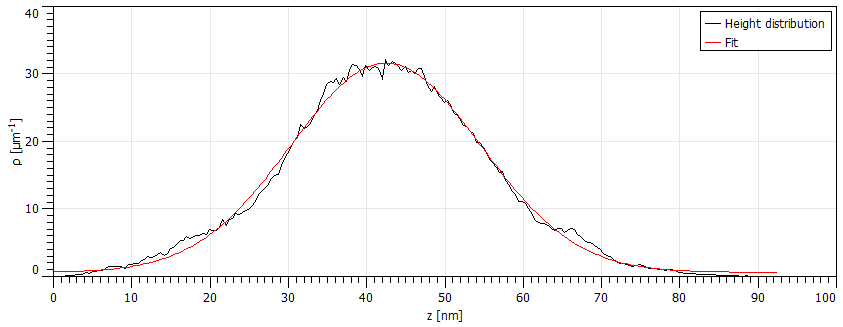


(a)


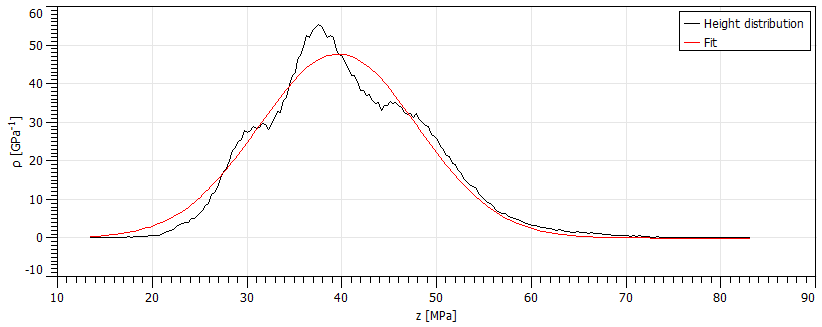


(b)


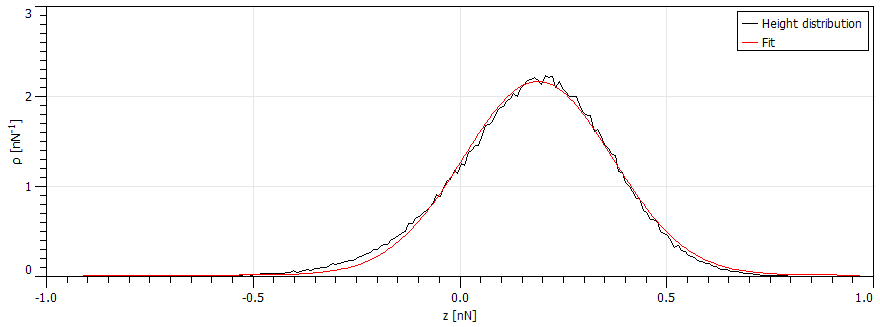


(c)

===== Fit Results =====

Data: Height distribution

Number of points: 223 of 223

X range: 0.000 to 92.285 nm

Fitted function: Gaussian

Results

x0 = 4.2444e-008 ± 6.11636e-011 m

y0 = 619609 ± 96003.7 m^-1

a = 3.10001e+007 ± 151599 m^-1

b = 1.71584e-008 ± 1.14262e-010 m

Residual sum: 6.31703e+011

Correlation matrix

1.000

-0.001 1.000

0.001 -0.451 1.000

0.001 -0.653 -0.096 1.000


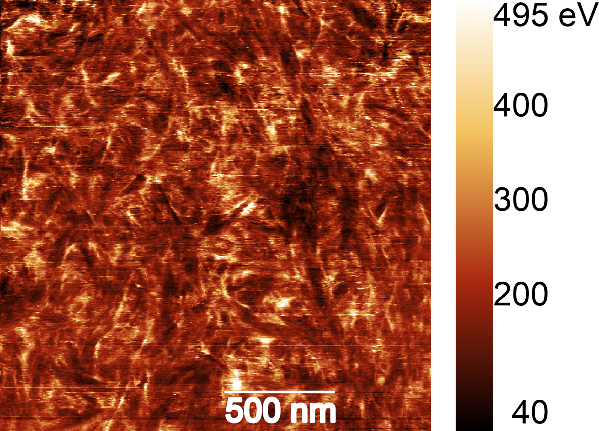

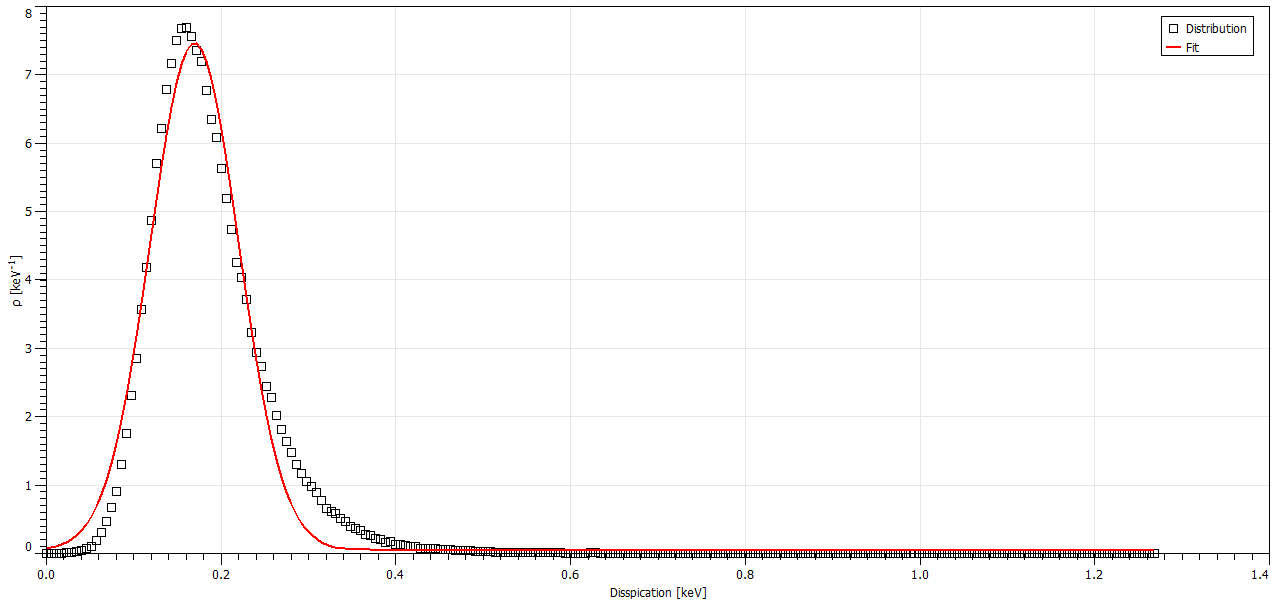


(d)

===== Fit Results =====

Data: Height distribution

Number of points: 223 of 223

X range: 0.0000 to 1.2684 keV

Fitted function: Gaussian

Results

x0 = 169.129 ± 0.644012 eV

y0 = 4.91379e-005 ± 1.99262e-005 eV^-1

a = 0.00741131 ± 8.31154e-005 eV^-1

b = 71.3825 ± 0.949798 eV

Residual sum: 7.00036e-008

Correlation matrix

1.000

-0.001 1.000

0.000 -0.171 1.000

0.000 -0.283 -0.497 1.000

**Fig S9**

The dissipation data was provided in Fig. 7 and discussed in the main text of the paper. To support the discussions regarding the loss of entropy assumption during the conformational changes of the surface of the nanocelluloses. Below is an example showing the fitted results of the dissipation map for a CNC sample measured in PBS 3.5, (image 003). The fitting was conducted using the function in Gwyddion as described in experimental section for modulus, adhesion force and height distribution.
