# Supplementary material for: Effect of decoration route on the nanomechanical, adhesive, and force response of nanocelluloses—An in situ force spectroscopy study
Source: PLoS One. 2023 Jan 3;18(1):e0279919. doi: 10.1371/journal.pone.0279919 (PMC9810197; doi:10.1371/journal.pone.0279919)
Supplement: S5 Fig — The logged FD curves of (a) CNC, (b) LCNC and (c) TCNF as displayed in the main text of Fig 6. The retraction force curves of the (d) CNCs, (e) LCNCs and (f) TCNFs plotted together with the approach curves are presented in Fig 6. Deflection error vs. Z distance (nm) curves measured on each of the marked single fibre of the three materials at pH 7.2 or 3.5. (DOCX) [file pone.0279919.s008.docx]

**Supplementary information (SI)**

**S11 Fig. The logged FD curves of (a) CNC, (b) LCNC and (c) TCNF as displayed in the main text of Fig 6. The retraction force curves of the (d) CNCs, (e) LCNCs and (f) TCNFs plotted together with the approach curves are presented in Fig 6. Deflection error vs. Z distance (nm) curves measured on each of the marked single fibre of the three materials at pH 7.2 or 3.5.**

The logged FD (logged(Force) vs. tip-surface separation (nm)) curves of (a) CNC, (b) LCNC and (c) TCNF and the retraction force curves of the (d) CNCs, (e) LCNCs and (f) TCNFs plotted together with the approach curves are displayed in Fig S11 below.

(b)

(a)

(d)

(c)

(f)

(e)


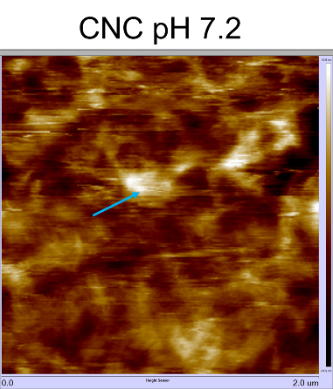

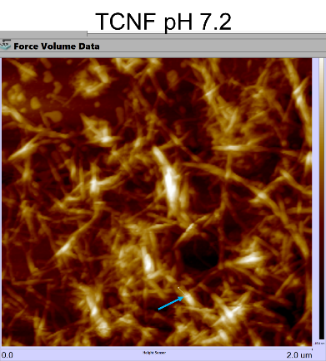

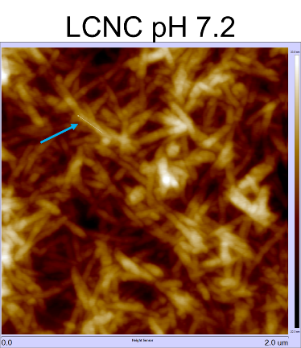


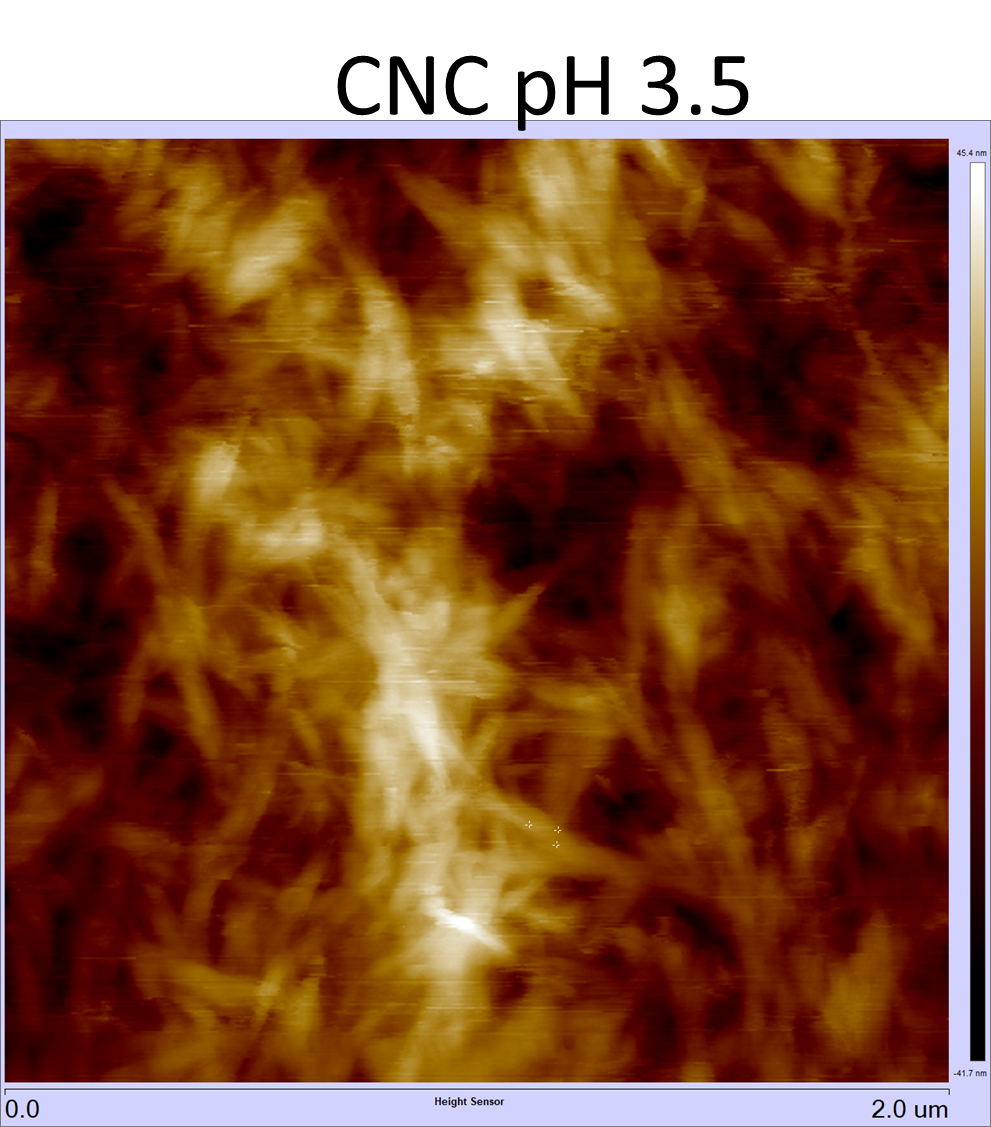

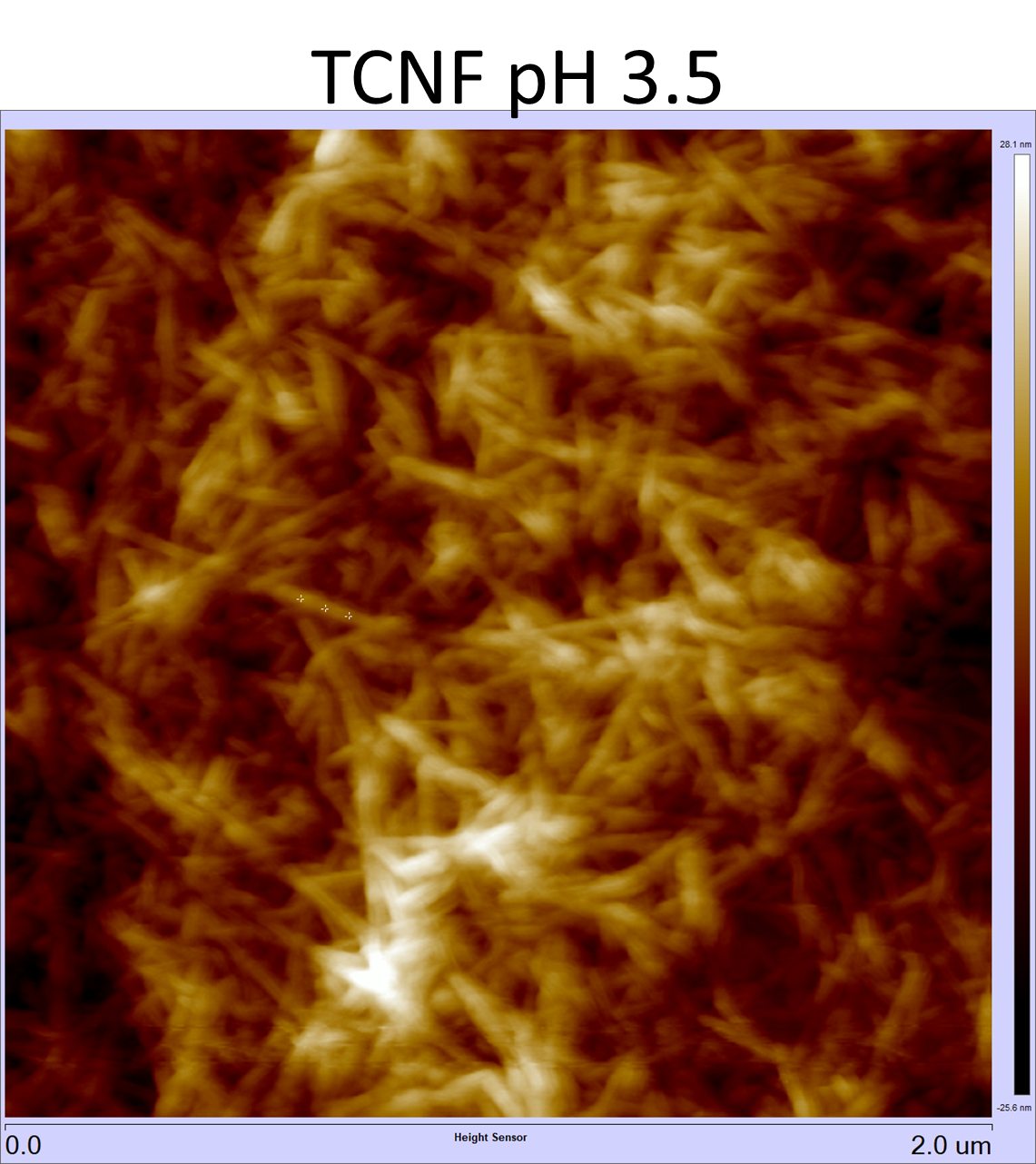

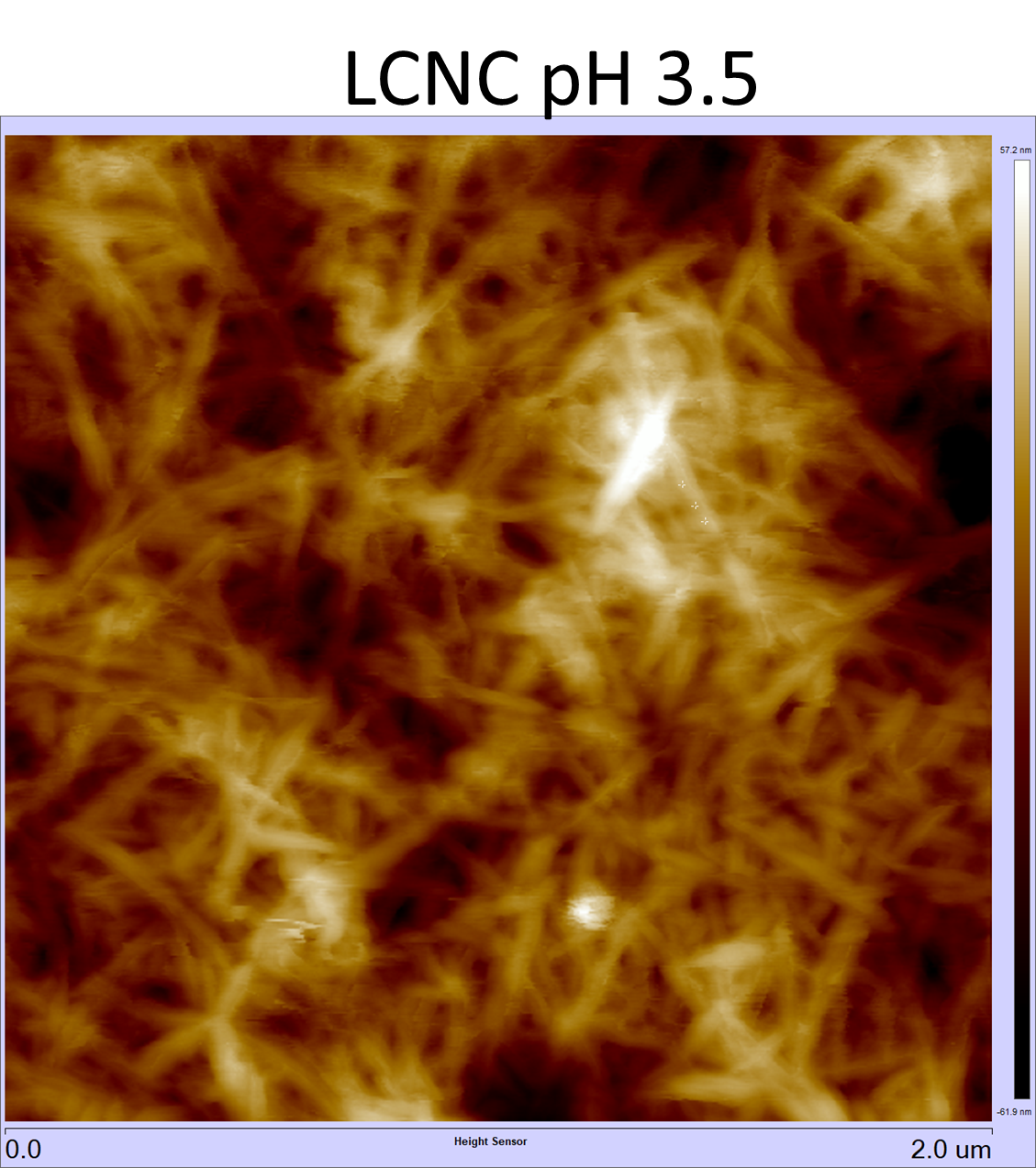


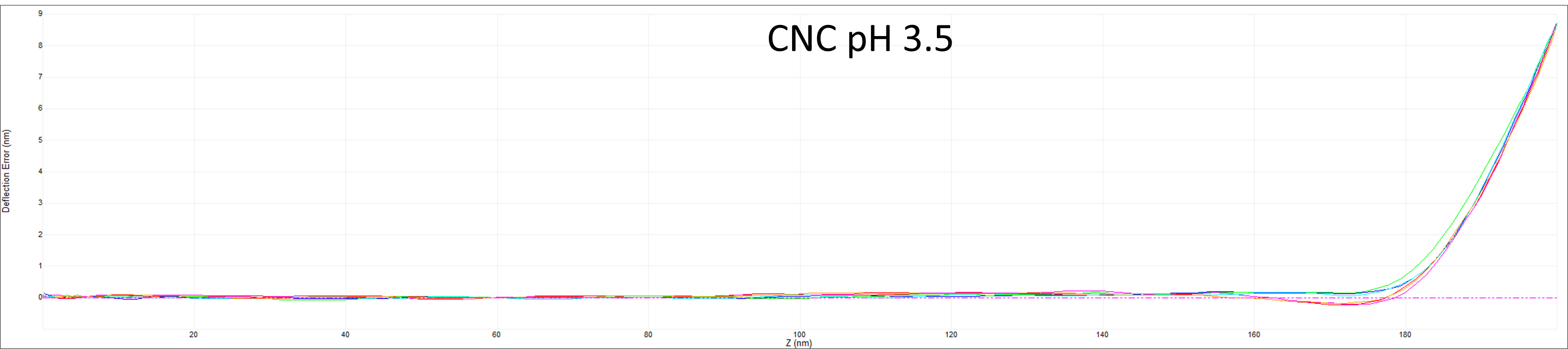


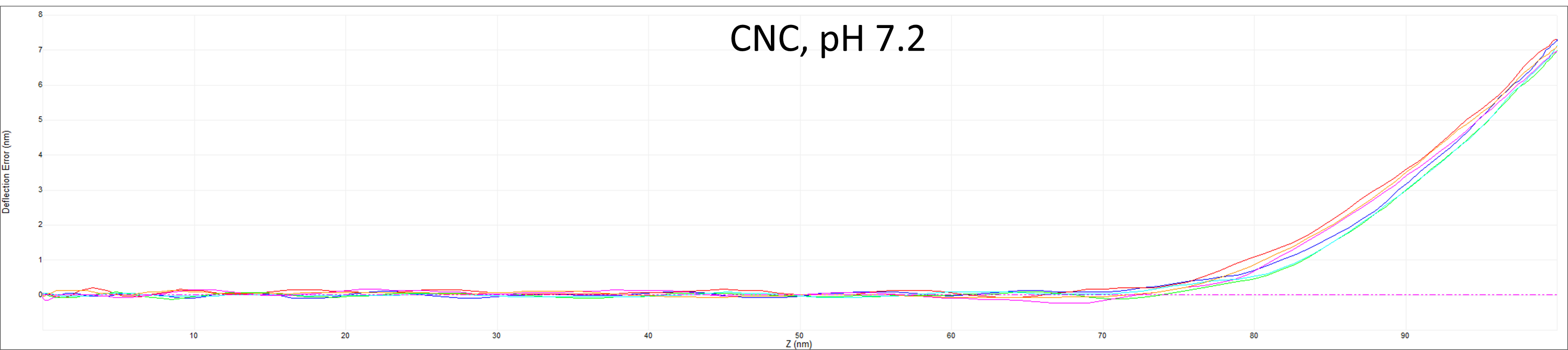


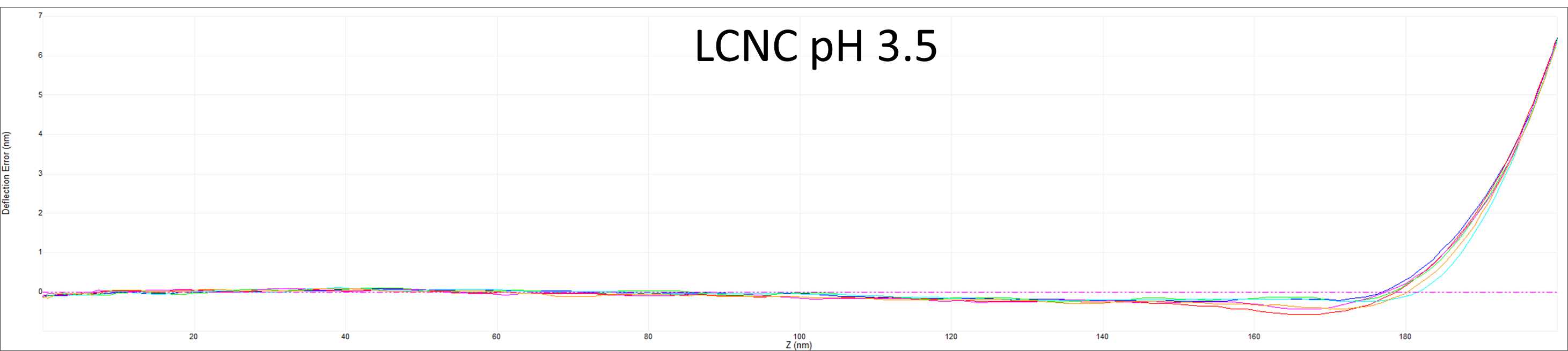


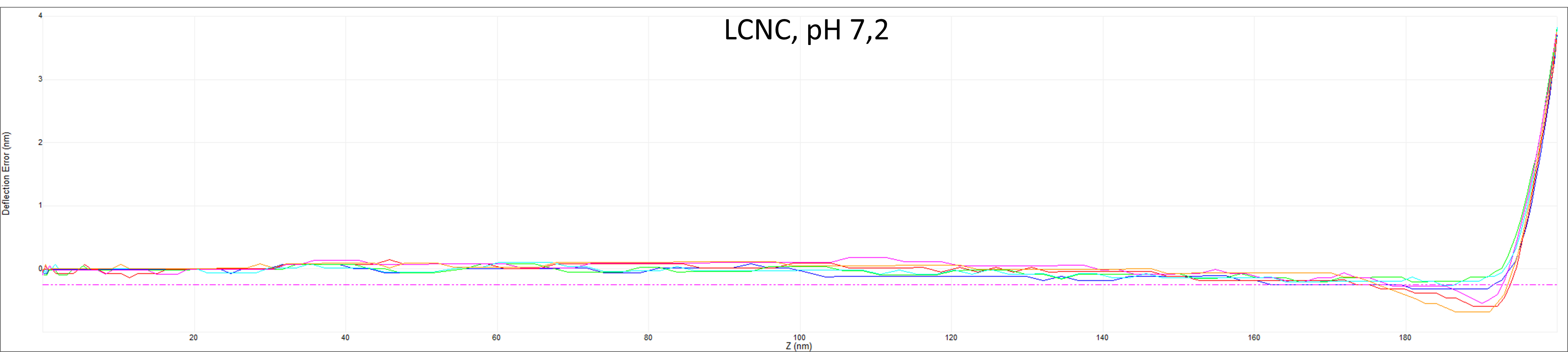


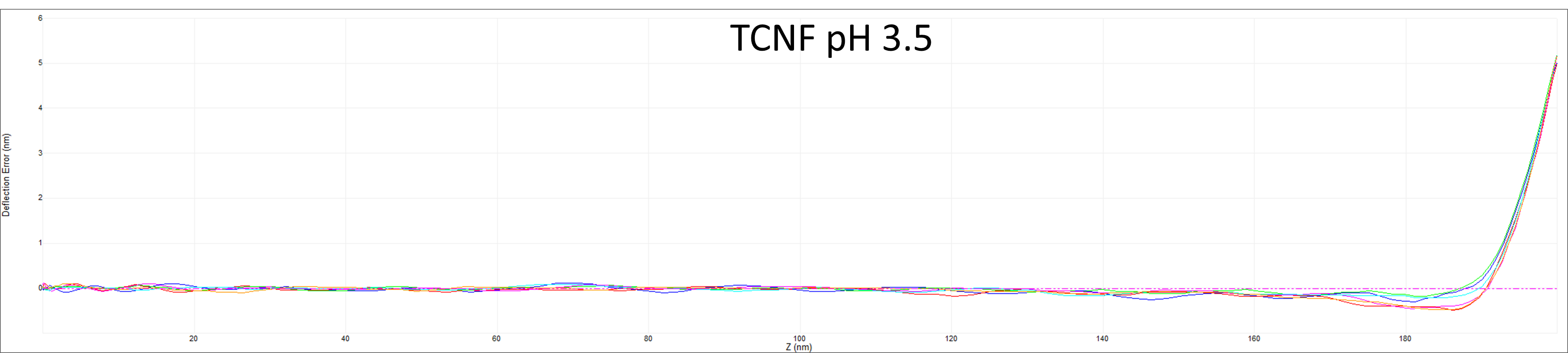


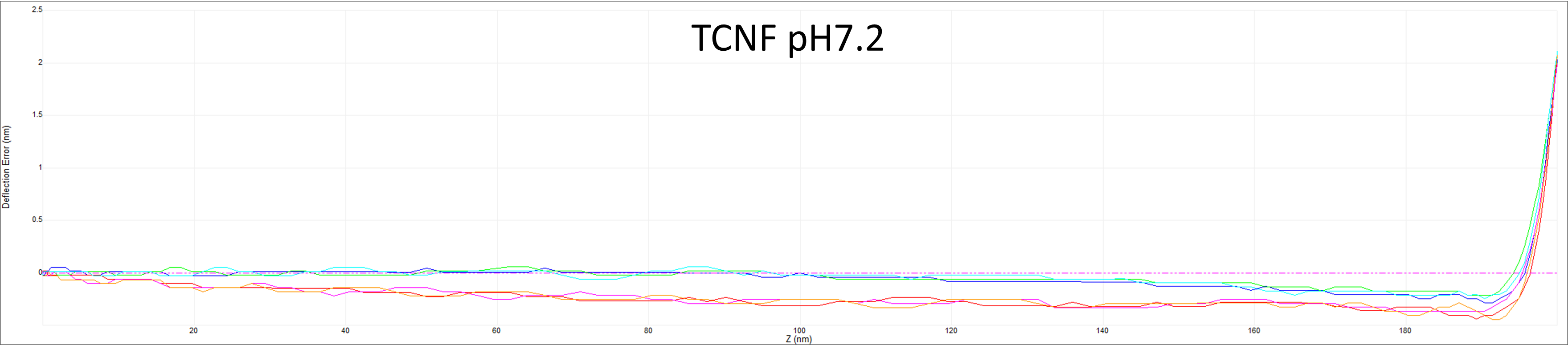


**Fig S11**

The plots shown in (d), (e), (f) are the retract curves shown in dash lines displayed together with the approach curves vs. tip-surface separation. In addition to the discussions in main text, we observed that hysteresis of the retracting curves of FD force for all three cases were observed in salt solutions, hysteresis rarely occurred in pure water. This is again due to the highly charged nature of the grafted groups at the nanocelluloses. Inter diffusion of the cellulose surfaces is likely adding to hysteresis in electrolytes, which is related to a non-equilibrium state of the charged polyelectrolyte group segment density in the gap of cellulose surfaces. [1] In addition, as we can see in the deflection error. vs. Z distance (nm) curves measured for a fibre by drawing cross line (as marked by blue arrow in the AFM images). The approaching curves are always more repulsive than those determined on tip separation. This hysteresis is strong evidence that the measured forces are mainly of steric origin.

**Reference**

1. Klein J, Luckham PF. Forces between two adsorbed poly(ethylene oxide) layers in a good aqueous solvent in the range 0-150 nm. Macromolecules. 1984;17: 1041–1048. doi:10.1021/ma00135a011
