# Supplementary material for: Effect of decoration route on the nanomechanical, adhesive, and force response of nanocelluloses—An in situ force spectroscopy study
Source: PLoS One. 2023 Jan 3;18(1):e0279919. doi: 10.1371/journal.pone.0279919 (PMC9810197; doi:10.1371/journal.pone.0279919)
Supplement: S6 Fig — Line profile of a randomly chosen area of each of the DMT modulus mapping (drawing a horizontal and a vertical crossing line over an image) and adhesion force distribution (drawing a line over a single fibre) of the PFQNM images of (a, a´) CNCs, (b, b´) TCNCs and (c, c´) LCNCs obtained in air and in PBS buffers. Representative line profiles of height and width of a single fibre of CNC, TCNF, LCNC obtained in ambient (air) condition, by randomly chosen five single fibres on the same sample. (DOCX) [file pone.0279919.s009.docx]

**Supplementary information (SI)**

**S13 Fig. Line profile of a randomly chosen area of each of the DMT modulus mapping (drawing a horizontal and a vertical crossing line over an image) and adhesion force distribution (drawing a line over a single fibre) of the PFQNM images of (a, a´) CNCs, (b, b´) TCNCs and (c, c´) LCNCs obtained in air and in PBS buffers. Representative line profiles of height and width of a single fibre of CNC, TCNF, LCNC obtained in ambient (air) condition, by randomly chosen five single fibres on the same sample.**

(a). Line profiles of DMT modulus and (a´) distribution of adhesive force at a single CNC fibre.

(b). Line profiles of DMT modulus and (b´) distribution of adhesive force at a single TCNF fibre.

(c). Line profiles of DMT modulus and (c´) distribution of adhesive force at a single LCNC fibre.


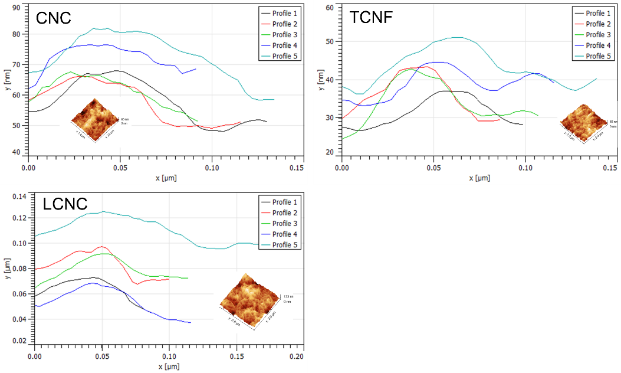


**Fig S13**
