# Supplementary material for: Effect of decoration route on the nanomechanical, adhesive, and force response of nanocelluloses—An in situ force spectroscopy study
Source: PLoS One. 2023 Jan 3;18(1):e0279919. doi: 10.1371/journal.pone.0279919 (PMC9810197; doi:10.1371/journal.pone.0279919)
Supplement: S1 Table — (DOCX) [file pone.0279919.s010.docx]

**Supplementary information (SI)**

**S4 Table: Information on the surface chemical properties of the nanocelluloses in PBS buffer solutions**

**Table S4**

| Type | Ions in medium | Zeta potential (mV) | pH | Surface groups | Counter ions | Contact angle (CA) (degree) |
| --- | --- | --- | --- | --- | --- | --- |
| CNC | PO_4_^3−^ | -17.0 ± 0.37 | 3.5 | OSO_3_^−^ | K^+^ | 34.25 ± 0.35 |
|  | PO_4_^3−^, Cl^−^ | -18.4 ± 0.85 | 7.2 | OSO_3_^−^ | Na^+^, K^+^ |  |
| TCNF | PO_4_^3−^ | -20.4 ± 1.52 | 3.5 | COO^−^ | K^+^ | 35.75 ± 0.14 |
|  | PO_4_^3−^, Cl^−^ | -25.3 ± 0.79 | 7.2 | COO^−^ | Na^+^, K^+^ |  |
| LCNC | PO_4_^3−^ | -10.7 ± 0.20 | 3.5 | Phenolic, CHO, COO^−^ | K^+^ | 34.22 ± 0.16 |
|  | PO_4_^3−^, Cl^−^ | -18.2 ± 7.43 | 7.2 | Phenolic, CHO, COO^−^ | Na^+^, K^+^ |  |
