# Supplementary material for: Effect of decoration route on the nanomechanical, adhesive, and force response of nanocelluloses—An in situ force spectroscopy study
Source: PLoS One. 2023 Jan 3;18(1):e0279919. doi: 10.1371/journal.pone.0279919 (PMC9810197; doi:10.1371/journal.pone.0279919)
Supplement: S2 Table — (DOCX) [file pone.0279919.s011.docx]

**Supplementary information (SI)**

**S5 Table: Representative values of the calibrated spring constant of ScanAsyst-Fluid cantilevers prior to each PFQNM measurement in PBS buffer;**

**Table S5**

| k (N\m) | CNC | LCNC | TCNF |
| --- | --- | --- | --- |
| pH3.5 | 1.169 | 0.9865 | 1.0874 |
| pH7.2 | 1.4937 | 1.0311 | 1.6711 |
