# Supplementary material for: Effect of decoration route on the nanomechanical, adhesive, and force response of nanocelluloses—An in situ force spectroscopy study
Source: PLoS One. 2023 Jan 3;18(1):e0279919. doi: 10.1371/journal.pone.0279919 (PMC9810197; doi:10.1371/journal.pone.0279919)
Supplement: S3 Table — (DOCX) [file pone.0279919.s012.docx]

**Supplementary information (SI)**

**S8 Table: Fitted values of distribution of the height, DMT modulus, and adhesion force mappings displayed in Figs 2 - 4 in the main text;**

**Table S8**

| **CNC** | **PFQNM** | **Height** | **DMT modulus** | **Adhesion force** |
| --- | --- | --- | --- | --- |
|  | air | 34.802nm± 30 pm | 126.47 Mpa ± 0.13 Mpa | 11.263 nN± 74 pN |
| pH 3.5 | 30 mins | 42.444nm± 61 pm | 36.634 Mpa ± 0.11 Mpa | 188.58 pN± 0.84pN |
|  | 180 mins | 43.05 nm ± 98 pm | 35.558 MPa± 97 Kpa | 267.74 pN± 1.2pN |
|  | air | 11.148nm ± 48 pm | 220.37 MPa± 0.62 Mpa | 2.6602 nN± 39 pN |
| pH7.2 | 30mins | 14.345nm± 67 pm | 42.937MPa± 75 kPa | 1.221 nN± 2.5 pN |
|  | 180 mins | 20.791nm ± 41 pm | 11.328MPa± 16 kPa | 858.69 pN± 1.1 pN |

| **LCNC** | **PFQNM** | **Height** | **DMT modulus** | **Adhesion force** |
| --- | --- | --- | --- | --- |
|  | air | 20.694 nm± 53pm | 188.85 MPa± 0.61 MPa | 6.5495 nN± 60 pN |
| pH 3.5 | 30 mins | 54.827 nm± 94pm | 31.712 MPa± 0.12 MPa | 1.4568 nN± 2.0 pN |
|  | 180 mins | 66.644 nm± 0.71nm | 9.4781 MPa± 44 kPa | 893.66 pN± 1.5 pN |
|  | air | 25.929 nm± 42 pm | 65.548 MPa± 94 kPa | 8.081 nN± 0.11 nN |
| pH7.2 | 30mins | 27.482 nm± 55 pm | 289.12 MPa± 1.5 MPa | 571.84 pN± 2.5 pN |
|  | 180 mins | 34.588 nm± 73pm | 66.475 MPa± 0.17 MPa | 830.52 pN± 0.84 pN |

| **TCNF** | **PFQNM** | **Height** | **DMT modulus** | **Adhesion force** |
| --- | --- | --- | --- | --- |
|  | air | 14.653 nm± 18 pm | 29.573 MPa± 56 kPa | 7.08 nN± 49 pN |
| pH 3.5 | 30 mins | 23.44 nm± 27 pm | 374.86 MPa± 0.73 MPa | 657.28 pN± 0.94 pN |
|  | 180 mins | 25.818 nm± 41 pm | 134.42 MPa± 0.54 MPa | 670.15 pN± 0.7 pN |
|  | Air | 43.287 nm± 92pm | 84.978 MPa± 61 kPa | 11.669 nN± 35 pN |
| pH7.2 | 30mins | 43.365 nm± 84pm | 27.711 MPa± 15 kPa | 1.416 nN± 1.2 pN |
|  | 180 mins | 32.613 nm± 73 pm | 52.125 MPa± 40 kPa | 1.5308 nN± 1.5 pN |
