# Supplementary material for: Effect of decoration route on the nanomechanical, adhesive, and force response of nanocelluloses—An in situ force spectroscopy study
Source: PLoS One. 2023 Jan 3;18(1):e0279919. doi: 10.1371/journal.pone.0279919 (PMC9810197; doi:10.1371/journal.pone.0279919)
Supplement: S4 Table — (DOCX) [file pone.0279919.s013.docx]

**Supplementary information (SI)**

**S10 Table: Root mean square roughness (*Rq*) values of the height mappings (500 nm × 500 nm) of PFQNM results as a function of immersion time;**

**Table S10**

| R_q (_ nm) | CNC | | TCNF | | LCNC | |
| --- | --- | --- | --- | --- | --- | --- |
|  | 30 mins | 180 mins | 30 mins | 180 mins | 30 mins | 180 mins |
| pH 3.5 | 17.2 ±1.4 | 13.1 ±0.5 | 19.1±10.2 | 30.3± 19.1 | 22.7± 8.6 | 27.7± 5.9 |
| pH 7.2 | 6.3 ± 0.0 | 5.6 ± 0.6 | 6.4 ±0.2 | 8.4 ±1.0 | 2.7 ± 0.1 | 2.6± 0.1 |
