# Supplementary figures and images for: Effect of decoration route on the nanomechanical, adhesive, and force response of nanocelluloses—An in situ force spectroscopy study
Source: PLoS One. 2023 Jan 3;18(1):e0279919. doi: 10.1371/journal.pone.0279919 (PMC9810197; doi:10.1371/journal.pone.0279919)

**Table of Contents/Abstract Graphics**


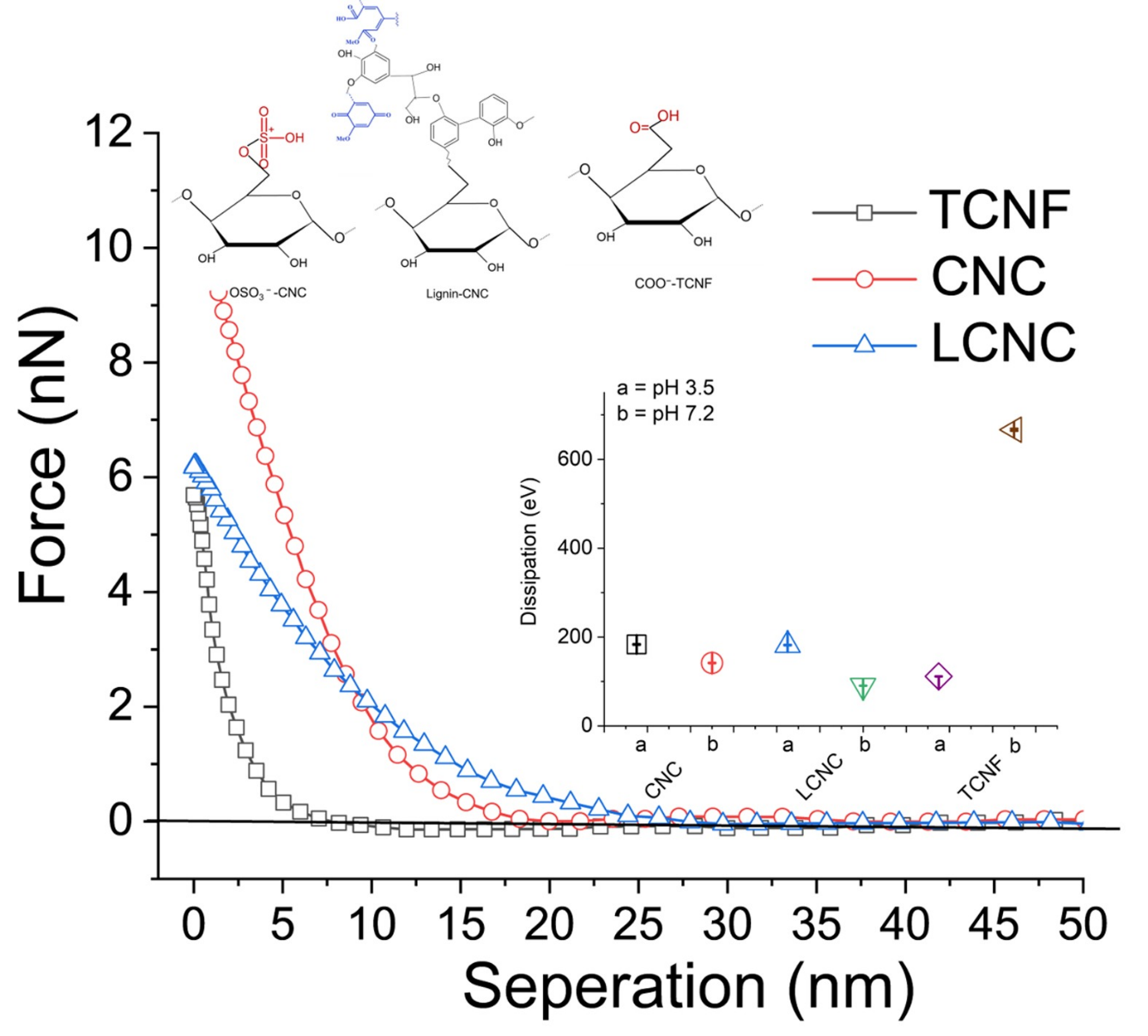

Supplement: S1 Graphic abstract — (DOCX) [file pone.0279919.s014.docx]
